# A New Opinion Polarization Index Developed by Integrating Expert Judgments

**DOI:** 10.3389/fpsyg.2021.738258

**Published:** 2021-10-13

**Authors:** Namkje Koudenburg, Henk A. L. Kiers, Yoshihisa Kashima

**Affiliations:** ^1^Social Psychology, Department of Psychology, Heymans Institute, University of Groningen, Groningen, Netherlands; ^2^Psychometrics and Statistics, Department of Psychology, Heymans Institute, University of Groningen, Groningen, Netherlands; ^3^Social and Cultural Psychology, School of Psychological Sciences, University of Melbourne, Melbourne, VIC, Australia

**Keywords:** opinion distribution, scale development, bimodality, dispersion, opinion polarization, expert judgments

## Abstract

Opinion polarization is increasingly becoming an issue in today’s society, producing both unrest at the societal level, and conflict within small scale communications between people of opposite opinion. Often, opinion polarization is conceptualized as the direct opposite of agreement and consequently operationalized as an index of dispersion. However, in doing so, researchers fail to account for the bimodality that is characteristic of a polarized opinion distribution. A valid measurement of opinion polarization would enable us to predict when, and on what issues conflict may arise. The current study is aimed at developing and validating a new index of opinion polarization. The weights of this index were derived from utilizing the knowledge of 58 international experts on polarization through an expert survey. The resulting *Opinion Polarization Index* predicted expert polarization scores in opinion distributions better than common measures of polarization, such as the standard deviation, Van der Eijk’s polarization measure and Esteban and Ray’s polarization index. We reflect on the use of expert ratings for the development of measurements in this case, and more in general.

## Introduction

Opinion polarization is increasingly an issue in today’s society. This is because the extent to which opinions are polarized in a group (or society) is seen to determine the likelihood that a dispute or conflict may arise in the group. Surprisingly, however, we lack good measures to assess opinion polarization. Its conceptualizations and measurements often focus on either the dispersion of attitudes or on the bimodality of an attitude distribution. Nonetheless, the choice of an indicator may not always be well-founded, and largely determines whether a polarization is identified. A valid measurement of opinion polarization would enable us to predict when, and on what issues, conflict may arise. The current study is aimed at developing and validating a new index of opinion polarization, by integrating the knowledge of 60 experts on polarization.

### Why Do We Need to Capture Opinion Polarization?

Opinion polarization can produce both unrest at the societal level, and conflict within small scale communications between people of opposing opinions and viewpoints. Importantly, such unrest and conflict can be further fueled by actual polarization between opinions, where the positions taken are incompatible. However, unrest and conflict may just as well be wrought in case it is merely *thought* that opinions are polarized. In fact, research shows that opinion polarization may be much less present than often assumed (DiMaggio et al., 1996; Hoffmann and Miller, 1997; Fiorina et al., 2005; Evans, 2009). However, the current political discourse often suggests otherwise – (populist) parties emphasize the difference between societal groups, and the incivility in political exchange gives the impression that these differences are hard to reconcile. Moreover, the highly emotional and often one-sided content on (social) media stimulates a polarized view of reality, which often exaggerates actual differences between opinion groups (Stroud, 2010; Levendusky and Malhotra, 2016). Although these perceptions may not be real, in the sense that they adequately reflect the extent to which opinions between groups differ, they are real in their consequences for emotions, cognitions and behavioral intentions (e.g., Wojcieszak, 2011; Koudenburg and Kashima, 2021).

The current research aims to develop an Opinion Polarization Index that is suitable for measuring both *actual* opinion polarization (e.g., by examining a distribution of many individuals’ opinions on a topic obtained by a poll) and individual *perceptions* of opinion polarization (e.g., by asking a single individual how he/she thinks opinions on a certain topic are distributed in society).

### Conceptualization of Opinion Polarization

#### Definition Clarity

In this paper, we examine opinion polarization as a *state*, which we define as the extent to which opinions on an issue are opposed. This should be distinguished from the *process* of polarization, referring to the movement of opinions on an issue in opposite directions (DiMaggio et al., 1996). In earlier social psychological theorizing (e.g., Myers and Lamm, 1976), the term polarization was used to describe the phenomenon of interacting groups shifting *collectively* toward an extreme end of an opinion spectrum. Here, we mean by polarization a situation in which the state of an opinion distribution is such that there are *opposing* viewpoints within an opinion spectrum.

#### Conceptualizing Opinion Polarization as Dispersion

Some existing measures of opinion polarization conceptualize polarization as dispersion, namely, the extent to which opinions are diverse and distant from one another. The most commonly used measure of dispersion is either the variance or standard deviation of a distribution (e.g., DiMaggio et al., 1996; Gay et al., 1996). These measures are suitable for measuring opinion polarization in the sense that they increase when any two randomly selected respondents are likely to differ in their opinions, and when more opinions are located toward the extreme ends of the scale. However, there are also some shortcomings of using the standard deviation for indexing opinion polarization.

One shortcoming of the use of the standard deviation to assess opinion polarization in the case of rating scales with a fixed number of categories, is that it is influenced not only by the dispersion of an opinion distribution, but also by its skewedness (Van Der Eijk, 2001). This is especially problematic for assessing dispersion in distributions where the mean is located near one end of the scale, because the few cases at the other end strongly contribute to the standard deviation (because of their large difference from the mean)^1^. To overcome this problem with the standard deviation as a measure of dispersion, Van der Eijk developed a new measure that disaggregates frequency distributions into component parts called layers. At the level of these layers, (dis)agreement is determined and weighted by the number of observations in the layer. This results in a polarization measure that ranges from 0 (perfect agreement) to 1 (perfect polarization).

**FIGURE 1:**
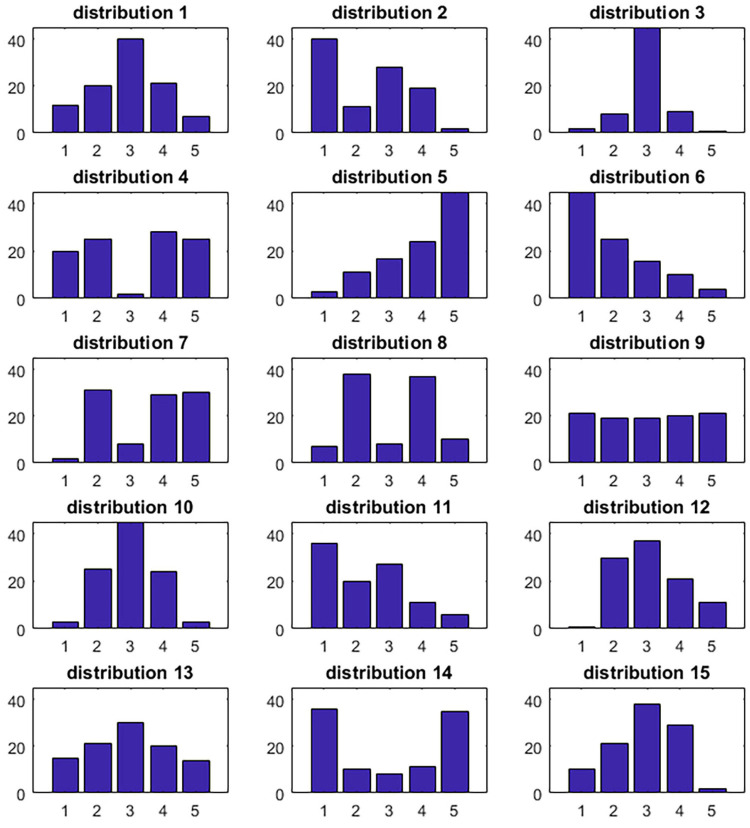
Distributions that were judged by 60 experts on the extent to which they represented a polarized state.

A second major critique of the standard deviation (e.g., Mouw and Sobel, 2001), which also applies to Van der Eijk’s measure, is that it treats ordinal scales as if they were interval scales. This means that the distance between an extreme opinion and a neutral opinion (say, 3 and 5 on a 5-point scale from disagreement to agreement) affects the standard deviation in the same way as two opinions that are located on contrasting sides of the scale (say, 2 and 4 on the same scale). In the case of opinion polarization, we deem it likely that the same distance may have different meanings depending on its location on a scale. We believe that a society in which a group of individuals with an extreme opinion is distinguishable from a group with a neutral opinion (e.g., Figure 1, distribution 2: [40 11 28 19 2], where numbers in square brackets indicate percentages of people who respond from 1 to 5) is unlikely to be perceived as polarized; however, polarization may be readily perceived when the opinions fall either on the “pro” or “con” side, even if these opinions are not extreme (e.g., Figure 1, distribution 8: [7 38 8 37 10]).

#### Conceptualizing Opinion Polarization as Bimodality

Now, in psychological theorizing, the focus has shifted from opinion differences to groups that represent these opinion differences. For instance, societal issues are increasingly discussed in terms of “Us” (those who share our opinion) vs. “Them” (those with an opposing opinion; McCoy et al., 2018), categorizing people into opinion-based groups (Abramowitz and Saunders, 2008; McGarty et al., 2009; Shor and McCarty, 2011). Psychologically, the understanding that another person does not only have an opinion different from one’s own, but that this opinion defines this person’s membership of a group whose defining characteristics are incompatible to one’s own, has a diverse range of consequences for one’s perceptions, feelings and behavioral intentions toward this person (Koudenburg and Kashima, 2021; see also Turner and Hogg, 1987). Specifically, when opinion differences within a community arise, this generally elicits some tension between its members (Schudson, 1997). By engaging in discussion people reduce this tension (Festinger and Thibaut, 1951; Schachter, 1951) while at the same time learning diverse information and political perspectives, thereby increasing their understanding of and tolerance toward others’ opinions (e.g., Gamson, 1997; Cappella et al., 2002; Mutz, 2006). The motivation to discuss disagreements, however, is significantly reduced when people believe an opinion difference is reflective of their belonging to incompatible groups. Instead, these beliefs elicit negative emotions, and motivate people to avoid the issue, encouraging even greater polarization of beliefs (Koudenburg and Kashima, 2021). Opinion polarization, thus, is not only reflected by a lack of agreement or by a high dispersion of opinions, but also concerns the extent to which people cluster into two opposing opinion groups. This theoretical shift toward recognizing the importance of the groupedness of opinions in predicting conflict suggests that when assessing opinion polarization, one should take into account the bimodality of a distribution (Esteban and Ray, 1994).

##### Empirical assessment of bimodality

Statistically, the clustering of people into separate opinion camps (i.e., the bimodality) has been assessed with the measure of Kurtosis (Walker and Lev, 1969; DiMaggio et al., 1996). Here, a topic on which consensus exists would have a peaked opinion distribution and therefore a positive kurtosis value. Kurtosis values below 0 would indicate a distribution that is flatter than a normal distribution. A completely bimodal distribution (with all responses equally spread at two different values) would be reflected by a kurtosis value of −2. Importantly, the measure of kurtosis is not affected by the distance between positions: when half of a population strongly disagrees and the other half is neutral on a position (say, scoring either 1 or 3 on a 5-point scale), kurtosis would be −2. This is the same kurtosis value as would be obtained when half of a population strongly disagrees, and the other half strongly agrees with a position (say, scoring either 1 or 5 on a 5-point scale). Theoretically, however, one would expect that the difference between positions would matter: A larger difference would make it more likely for people to categorize opponents into a different (opinion) group, and hence, increase chances that conflict occurs (Turner and Hogg, 1987; Miller and Hoffmann, 1999).

Econometric measures of *income* polarization (Wolfson, 1997; Duclos et al., 2004; Foster and Wolfson, 2010; Esteban and Ray, 2012) address this issue with kurtosis. Many (Wolfson, 1994; Foster and Wolfson, 2010) are not appropriate for opinion polarization because they indicate the extent to which people are distributed away from the median income. Although the median is a highly meaningful concept for income distribution (i.e., 50 percentile of the population, or the “middle class”), it lacks a theoretical meaning in opinion distribution. In political discourse, a more meaningful “middle ground” is a neutral point (or “neither agree nor disagree”) of two extreme viewpoints. Yet, the median of an opinion distribution does not necessarily mean the middle ground between two extreme viewpoints.

However, Esteban and Ray’s highly influential index of income polarization (1994; also see Duclos et al., 2004) is free of this limitation and its underlying conceptualization is potentially applicable to opinion polarization. According to them, polarization is the sum of all *effective antagonisms* felt by an individual toward others in a population. An effective antagonism is a perceiver’s negative attitude (or tension felt) toward a target person, which is thought to increase as a function of their *identification* with their ingroup and *distance* between the perceiver and the target on a given dimension. Esteban and Ray assumed that a perceiver’s identification increases as their ingroup size increases, and the distance can be measured by the absolute difference between their positions on the relevant dimension. As we will explicate later, we propose an empirically parameterized measure of opinion polarization, the Opinion Polarization Index, that is conceptually similar to Esteban and Ray’s, but with two differences that make it suitable for measuring opinion polarization, that is, introducing a meaningful midpoint and interpreting absolute polarization scores (normalization of scores between [0,100]).

#### Conceptualizing Opinion Polarization as a Dichotomy

With regard to voting, for instance, polarization is often conceptualized as taking sides when there are two opposing candidates, stances, or viewpoints. Such dichotomies have also been used to back up the claim that societies are ultimately divided. When conceptualizing polarization as a dichotomy, the percentage of people in a population supporting each position becomes an indication of opinion polarization: the more equally divided the opinions are, the higher opinion polarization (Baker, 2005). The measurement has been criticized to lead to an overestimation of opinion polarization: as respondents have to choose one or the other, they are forced into a polarized answering pattern (Demerath and Yang, 1997).

### The Current Approach

Rather than using measures that have been based on a particular reasoning on what to take into account, the present research takes an empirical approach to assessing opinion polarization that could account for both the clustering of the data into two (or more) groups, the distance between these groups, and other aspects of an opinion distribution that might contribute to polarization. We propose to develop a new Opinion Polarization Index (*P*) to predict the amount of opinion-based psychological distance, dispute or conflict in a population. Assuming that an opinion is measured on an *m*-point scale, and a position on the scale is indicated by an integer *i* or *j* (1 ≤ *i*, *j* ≤ *m*), Opinion Polarization Index can be written as below:


$$P=\sum_{j=1}^{m}\sum_{i=1}^{m}b\,\left(i,j\right)\,f\,\left(i,j\right),$$

where *b(i,j)* represents the *psychological distance* from position *i* to position *j*, and *f(i,j)* represents the frequency of all pairings of individuals at position *i* with the individuals at position *j* (*i* ≠ *j*).

This index is a weighted average of all possible pairs of psychological distances given an opinion distribution. For example, if we employ an opinion scale with the values 1,…, 5, there are 15 possible pairs of scores (1,1), (1,2),…, (5,5). They can be seen to represent all possible pairwise interactions between two individuals with opinions along the opinion spectrum from 1 to 5. The index assumes that an individual with position 1, for instance, experiences a *psychological distance* from another individual with position 5 at *b*(*1,5*). More generally, an individual whose opinion is at position *i* is thought to experience an opinion-based psychological distance of *b*(*i,j*) to another individual with an opinion at position *j*. *f*(*i,j*) is the frequency of pairings of people with opinion *i* and opinion *j*. In combination, the index reflects a sum total of psychological distances experienced within the population.

This index takes a similar starting point as the Esteban-Ray index (1994) in that it conceptualizes polarization as a sum total of opinion-based differentiation within the population. Esteban and Ray called this effective antagonism, which is a function of the absolute distance between two positions (i.e., |*i*–*j*|). In contrast, we suggest that this differentiation should be conceptualized as a *psychological* distance, and may need to be scaled differently with regard to the neutral middle ground on the opinion spectrum. For instance, (1,2) and (2,3) both differ one point and are on the same side of neutral point, 3; (1,3) and (2,4) both differ two points, but the first has scores on the same side of the neutral point, whereas the second has scores on different sides of the neutral point. In this way one can define different types of score pairs characterized by degree of dispersion and by whether or not they are on the same side of the neutral point. The weight of a pair, *b*(*i,j*), may differ depending on whether *i* and *j* are located on the same side or opposite sides of the neutral point. Put differently, the weight may be construed as a perceived likelihood of dispute or conflict if they interact. We assume *b(i,j)* = *b(j,i)* in this paper. We next count how often each pair of scores is present in a distribution, and define an index of opinion polarization in terms of these frequencies, with different weights attached to different *types* of score pair frequencies. That is, we will assume that the same weights will be given to pairs of values that have the same distance, provided they are on the same side of the neutral point. E.g., the weights for (1,2), (2,3), (3,4), and (4,5) will be equal. So will be those for (1,3) and (3,5) with a distance of two between values on the same side of neutral, but these need not be equal to that for (2,4; with a distance of two between values at different sides of neutral).

Of course, the method crucially depends on the choice of the weights, *b*(*i*,*j*). In order to estimate the weights, we consulted experts in the field of research on opinion polarization. Rather than merely reasoning what to us would seem a proper set of weights, we aimed to empirically estimate the appropriate weights to best predict experts’ opinion polarization judgments. We approached researchers who studied opinion polarization in different fields (e.g., political science, sociology, and psychology) and asked them to judge the extent of polarization of a set of 15 opinion distributions on a five-point scale. By using regression analyses, estimates were obtained for the weights to compute an Opinion Polarization Index that best predicted the actual polarization scores by the experts. Subsequently, we compared this Opinion Polarization Index to three existing measures of polarization: the Normed Standard Deviation, Van der Eijk’s measure of Polarization, and the Esteban-Ray index, and evaluated their abilities to predict the expert judgments of opinion polarization.

The aim of the current research was two-fold: First and foremost, we aimed to develop an index for assessing opinion polarization that did not rely on a single theoretical framework, but rather used a bottom-up approach which quantitatively integrates expertise on opinion polarization from the different fields of study. Second, we analyzed qualitative descriptions of polarization to identify aspects that experts deemed important in defining polarization. These descriptions would give insight into what qualities of opinion distributions experts took into account in their judgments, which were the basis of the Opinion Polarization Index.

## Materials and Methods

### Selection of Experts

We used the Web of Science search engine to identify articles published between January 2015 and May 2019 with “polarization” as a keyword. We restricted the search to the categories of social psychology, international relations, sociology and political science which yielded 1,157 results. We narrowed down this number by selecting papers of authors that had published at least 2 papers on polarization, yielding 391 papers. We invited the first two authors whose email addresses were provided in the Web of Science to partake in the survey. Often only the email address of the first author was provided, sometimes also of a co-author. Note that this means that not all invited authors had published 2 or more articles, because we included articles of which one author had published 2 or more articles. However, this strategy would mean that a large proportion of authors included in the study had published more than one paper on the topic, and were therefore more likely to be considered an expert. We finally invited 294 authors to partake in the survey via email.

Sixty authors participated in the survey (response rate 20.4%). Experts represented different subject areas, with a majority in political science (*n* = 47), and the others in psychology (*n* = 2), international relations (*n* = 2), sociology (*n* = 4), communication science (*n* = 3), and economics (*n* = 1). They indicated studying the topic for more than 10 years (*n* = 16), 3--10 years (*n* = 28), or less than 3 years (*n* = 9)^2^. The experts had academic positions ranging from PhD-student (*n* = 1), Post-doctoral researcher (*n* = 5), assistant professor (*n* = 20), associate professor (*n* = 15), to full professor (*n* = 18). One respondent did not complete the demographics. The study was approved by the Ethics Committee Psychology of the University of Groningen, approval code: PSY-2021-S-0154. Experts provided written consent before participation.

### Survey

Experts were asked to judge the extent to which they thought histograms of 15 opinion distributions represented a polarized state. Each graph displayed a total of 100 opinions distributed on a scale from 1 to 5, referring to opinions that ranged from “Strongly disagree,” “Disagree,” “Neutral,” “Agree,” to “Strongly agree.” To help them calibrate their judgments, we provided graphs of three example distributions, two of which represented an Opinion Polarization Index score of 0: Example A [100 0 0 0 0], and Example B [0 0 100 0 0], and one that represented an Opinion Polarization Index score of 100, Example C [50 0 0 0 50]. We explained that, although opinion polarization is sometimes defined to occur when opinions shift to one of the extreme ends of the distribution, we defined a polarized state for the purposes of the current study as the degree of dividedness within the distribution. Following this definition, we explained that Example A received an Opinion Polarization Index Score of 0. After the examples, experts continued by judging the 15 opinion distributions displayed in Figure 1 by giving each a score ranging from 0 = not at all polarized, to 100 = completely polarized.

Afterward, we asked their primary field of research, the number of years they had studied the topic of polarization, and their current position. We also asked experts to indicate in three open-ended questions what theoretical framework they used for studying polarization, how they came to their judgments for the distributions (e.g., what aspects of the graphs they took into account), and how they defined polarization. Finally, we asked whether they had any further comments.

## Results

### Qualitative Analyses

We conducted a thematic analysis on the answers to the open questions to identify the themes that experts deemed relevant for their assessment of polarization. We used an inductive approach, in which themes were first identified, and then two of the authors coded whether each of these themes was present in the answers of 44 experts who answered the open-ended questions (14 experts did not answer the open-ended questions, and the answers of two experts were deemed uncodeable). We used an iterative procedure to come to a shared understanding of the themes. This procedure increased the reliability of the coding. Four themes were identified: Clustering, Distance, Extremity, and Balance. Cohen’s Kappa for each theme ranged between 0.686 and 0.861, suggesting substantial (0.61–0.80) to almost perfect (0.81–1.00) interrater agreement (Landis and Koch, 1977). While the identified themes did not guide the development of our Opinion Polarization Index, they provide an insight into the aspects that were considered by the experts when giving their judgments on the distributions, and these judgments ultimately formed the basis of our index.

#### Clustering

Seventy-seven percent (82%) of the experts mentioned that opinion clustering (i.e., opinions are clustered into two or more clusters or groups) as a basis of their polarization judgment. The number in parentheses indicates the percentage of responses that one of the raters coded as reflecting this theme. Although a few experts suggested there may be more than two groups, a large majority suggested polarization implies two groups or clusters. In line with this, experts refer to the statistical concepts of bimodality, and the kurtosis of a distribution. Note that clustering does not provide information on the position of these groups with regard to each other. For example:

*“The presence of two internally homogeneous and completely separated groups (I actually believe the distance between groups is merely a matter of scale, so not so relevant per se*)*.”*

*“Distinct and homogeneous opinions.”*

#### Extremity

Forty-three percent (59%) of the experts mentioned that polarized groups, people, or opinions are located on the extreme ends of the scale. Sometimes, experts explicitly mentioned that very few moderate opinions exist: a hollow middle (note that, in this case, extremity also implies that opinions are clustered), but this is not always implied. An example of extremity:

*“In my view, polarization means few moderates/centrists, with attitudes concentrated in the tails of the distribution.”*

#### Distance

According to 39% (50%) of the experts, polarization implies that opinion groups are distant from one another. Or, when not explicitly mentioning groups, experts suggested that opinions are spread or dispersed across the distribution. Note that distance does not provide information about where opinions are located on the scale. An example of distance:

*“I also define it as the distance between opinions –, i.e., polarization requires two sides and a distance between them.”*

#### Balance

Only 16% of the experts (23%) stated that polarization implies there are equal proportions of people in each group (or on each side of the scale, or on each pole). We also coded for balance when experts mentioned that groups on either side should be at least “substantial.” For example:

*“A situation in which there are two distinct poles in which there are equal proportions of the population.”*

*“Substantial percentages of people on opposite sides of a scale.”*

#### Remaining Category

In addition to these four main themes, there were also characteristics mentioned that could not be assessed from a (single) opinion distribution. For instance, experts mentioned the strength of the attitudes, the extent to which they were moralized, or the importance of its content. They also underlined the importance of the relation of attitudes to group ideology, or affect. Although these are important aspects of polarization, we will not go into these here, because our focus is on polarization that can be derived from opinion distributions.

#### Conclusion

Based on the qualitative analyses, we identify four main themes that the experts considered central to opinion polarization, and used as a basis for judging the opinion distributions. While these themes have appeared in previous theorizing and measures on polarization, the Opinion Polarization Index is directly derived from the expert judgments, and our qualitative data therefore uniquely embodies all four themes. Indeed, the way the Opinion Polarization Index is conceptualized also reflects these qualitative ideas. For instance, scores on the Opinion Polarization Index are higher when there is a greater distance between clusters, when clusters are located at more extreme positions, and when clusters are more balanced.

### Quantitative Analyses

We started by screening the data for suspicious cases (section “Data Screening”) and conducting exploratory analyses to identify potential clusters in the data (section “Exploratory Analyses”). We then used regression analyses to obtain estimates for the weights for the Opinion Polarization Index that best predicted the actual polarization scores by the experts. We estimated these weights in two ways: First, we ignored pairs of scores on the same side of a distribution (section “Estimating the Opinion Polarization Index Weights”), as we assumed that these pairs would not contribute to polarization. Second, we checked this assumption by including these pairs into the regression analyses (section “Including Pairs With Scores on the Same Side of the Distribution”). Finally, we compared this Opinion Polarization Index to three existing measures of polarization: the Normed Standard Deviation, Van der Eijk’s measure of Polarization, and the Esteban-Ray index, and evaluated their abilities to predict the expert polarization judgments (section “Comparison With Other Polarization Indices”). All data and the full analysis procedure are given in the Supplementary Materials. We also provide an easy computational formula and an excel-sheet that researchers can use to calculate the Opinion Polarization Index.

#### Data Screening

We identified two suspicious cases, while screening the data. One expert scored all distributions on a range between 0 and 4 on the 100-point Opinion Polarization index. The ratings of a second expert correlated negatively with all but one of the other experts, and most of them were quite strongly negative. Because we think these experts are likely to have misunderstood the scale, their ratings were not included in further analyses, which were hence based on 58 experts.

#### Exploratory Analyses

To check whether we could identify clusters of experts that would score the distributions in a similar way, we used 2-dimensional MDS on Euclidean Distances (Borg and Groenen, 2005) between the 58 rows (using an MDS program obtained from Patrick Groenen). This gave a good fit, and no clear clustering in the response patterns was identified: the stress value was 0.045, and the fit percentage 95.5%.

#### Setting Up the Predictor Matrix

The distributions were presented in the form of histograms (see Figure 1). These were originally generated manually and chosen to have total frequencies equal to 100, but due to inadvertent truncation in the plots, this is not always the case. In distribution 3, the highest plotted frequency was 44, while 80 was intended, and in distributions 5, 6, and 10, the highest plotted frequency was 44, while 45 was intended. In those cases, we e used the actually plotted frequencies but next made them comparable to the others by dividing them by their sum and multiplying them by 100, so that all distributions had totals of 100.

As mentioned above, our goal is to develop a new Opinion Polarization Index based on weighting the frequencies of all possible pairs of scores that can occur for individuals. As a first step, we must for each of the 15 distributions, identify those frequencies of pairs of scores. We could consider all possible score pairs separately, but we decided to group them in equivalent pairs, and count these, as follows. For each distribution, we calculated

*f*_0_ = the frequency of pairs of equal scores
*f*_1_ = the frequency of pairs of scores differing by 1 point
*f*_2_*_*a*_* = the frequency of pairs of scores (1,3) and (3,5), hence differing by 2 points on the same side
*f*_2_*_*b*_* = the frequency of pairs of scores (2,4), hence differing by 2 points on different sides
*f*_3_ = the frequency of pairs of scores differing by 3 points
*f*_4_ = the frequency of pairs of scores differing by 4 points.

Note that score pairs (*i,j*) and (*j,i*) have both been counted, even though obviously they are equivalent. Score pairs (*i,i*), that is, of an individual with him or herself have been discarded.

After counting the score pairs, the counts were converted into percentages by dividing by the total number of pairs (=100^2^–100 = 9,900) and by multiplying by 100, just to make them palatable numbers in the same range as the agreement scores by the experts.

Thus, for each of the 15 distributions, we have scores on 6 possible characteristics of opinion polarization. These scores actually are *percentages* of comparisons that fall into each of the six categories defined (see Supplementary Material for an overview of the percentage of score pairs in each category per distribution). The goal of the present study is to assess how these should be weighted to get an opinion polarization measure that optimally predicts the expert judgments.

We tried to set up the distributions such that they vary widely in characteristics, and such that the characteristics themselves have substantively different contributions. To see to what extent this was successful, we inspected the correlation matrix (Table 1). There were some strong correlations that could destabilize regressions, involving *f*_1_ and *f*_2_*_*a*_*. This will be considered later on.

**TABLE 1:** Correlations between the different predictors of the Opinion Polarization Index.

	***f*_0_**	***f*_1_**	***f*_2_ *_*a*_***	***f*_2_ *_*b*_***	***f*_3_**
*f*_0_					
*f*_1_	0.20				
*f*_2_ *_*a*_*	–0.31	–0.03			
*f*_2_ *_*b*_*	–0.14	–0.05	–0.67		
*f*_3_	–0.60	–0.76	–0.02	0.20	
*f*_4_	–0.28	–0.66	0.03	–0.32	0.45

#### Estimating the Opinion Polarization Index Weights

Our goal now is to find weights for the above defined characteristics of opinion polarization such that we can define an Opinion Polarization Index that lies between 0 and 100 and best predicts the expert assessments of the degree of polarization for each of the 15 distributions. Specifically, we define the Opinion Polarization Index as the weighted sum of percentages *f*_0_,…, *f*_4_, by taking into account the requirement that it lies between 0 and 100, and by making an assumption on what should actually contribute to opinion polarization. We did that as follows.

First, consistent with our qualitative findings about the importance of extremity, we assumed that only *f*_2_*_*b*_*, *f*_3_, and *f*_4_ should contribute to opinion polarization (because these count pairs with scores on different sides of the neutral point), and that these differentially contribute to opinion polarization, while the others *f*_0_, *f*_1_, and *f*_2_*_*a*_* all do not contribute to opinion polarization and will hence be ignored. In section “Including Pairs With Scores on the Same Side of the Distribution,” we checked this assumption by conducting an analysis in which we did take *f*_0_, *f*_1_, and *f*_2_*_*a*_* into account, and it turned out that the resulting estimates led to the very same Opinion Polarization Index. So now we define our Opinion Polarization Index *P* as


(1) $$\text{P}={\text{b}}_{2}\,{\text{f}}_{2\,b}+{\text{b}}_{3}\,{\text{f}}_{3}+{\text{b}}_{4}\,{\text{f}}_{4}$$

where *b*_2_, *b*_3_, and *b*_4_ pertain to the weights we wish to assess. We have two requirements for our Opinion Polarization Index: *P* should at least be 0 and should not exceed 100. In Supplementary Material 1.5.1 we derive that this implies that *b*_4_ must equal 1.98, so that our general expression becomes


(2) $$\text{P}={\text{b}}_{2}\,{\text{f}}_{2\,b}+{\text{b}}_{3}\,{\text{f}}_{3}+1.98\,{\text{f}}_{4}$$

and that *b*_2_ and *b*_3_ will not be allowed to be negative, and should not exceed 1.98.

Using the above general expression for the Opinion Polarization Index, we want to estimate the free weights *b*_2_ and *b*_3_ such that the formula optimally predicts the average expert judgments for the 15 distributions. We do this by means of a variant of least squares regression, where one regression weight is fixed, and no intercept is being used. In other words, for each of the 15 distributions, we model the average expert judgments (*P*_*obs*_) as *P*_*obs*_ = *b*_2_*f*_2_*_*b*_ + b*_3_*f*_3_ *+* 1.98 × *f*_4_ + *e*, where *e* denotes an error term, and we search the weights *b*_2_ and *b*_3_ such that we minimize the sum of squared error terms over the 15 distributions. Because we expected that the optimal weights would satisfy the requirements, we used the unconstrained regression procedure without intercept. If it would have happened that the weights would either exceed 1.98, or become negative, we would have redone the analyses with constraint optimization procedures (e.g., see Lawson and Hanson, 1974).

The resulting weights were *b*_2_ = 1.07, with bootstrap 96% CI [0.93, 1.20], and *b*_3_ = 1.35, with bootstrap 95% CI [1.19, 1.51]. We computed bootstrap confidence intervals by resampling complete sets of judgment scores from the set of experts (see Supplementary Materials for scripts). Using this, the resulting formula for *P* would be

(3) $$\text{P}=1.07\,{\text{f}}_{2\,b}+1.35\,{\text{f}}_{3}+1.98\,{\text{f}}_{4}$$

For computational purposes, we can express^3^ this as


$$P=\left(2.14\times n_{2}\,n_{4}+2.70\times \left(n_{1}\,n_{4}+n_{2}\,n_{5}\right)+3.96\times n_{1}\,n_{5}\right)/\left(0.0099\,n^{2}\right),$$

where *n*_*i*_ (*i* = 1,…,5) denote the score frequencies, and *n* their sum. Incidentally, it should be noted that this formula satisfies our requirement that the minimal value of *P* is 0: This is because the three weights are positive, and the percentages used in this formula can never be negative, so *P* can never become negative, and hence the minimal value is 0.

We did the same analysis without distribution 3, and then found *b*_2_ = 1.06, *b*_3_ = 1.35, which suggests that the presence of distribution 3 does not seriously impact *P* in this analysis; because 3 does seem a valid representative of nonpolarized cases, we decided to keep it in our analyses.

#### Model Fit

**FIGURE 2:**
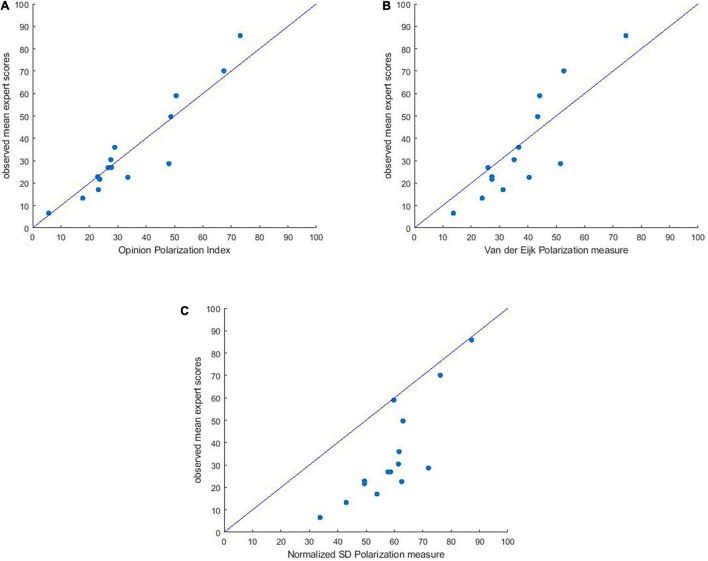
**(A)** Observed mean judgment scores for the 15 distributions plotted against the polarization values predicted by the Opinion Polarization Index, according to *P* = 1.07 × *f*_2_*_*b*_ +* 1.35 × *f*_3_ *+* 1.98 × *f*_4_. **(B)** Plot of mean expert polarization scores against the normalized standard deviation values for all 15 distributions. **(C)** Plot of mean expert polarization scores against Van der Eijk’s measure for all 15 distributions.

The resulting predicted values had a good fit with the average judgment scores: proportion unexplained sum of squares = 0.035 (96.5% fit), bootstrap 95% CI [0.022, 0.056]. That is, the model fit is 96.5%. In Figure 2A, it can be seen that predictions relatively speaking do not differ much from the average expert judgments. As a concrete measure of this (mis)fit we computed the Root mean squared error of estimation (RMSEA) = 7.57, bootstrap 95% CI [6.11, 9.49]. This value indicates that a “representative” difference between the average judgment scores, and the values predicted by our formula for *P* is 7.57 (representing the mean vertical distance of the points to the line in Figure 2A).

#### Including Pairs With Scores on the Same Side of the Distribution

As mentioned above, in developing our Opinion Polarization Index, we decided to ignore score pairs with scores on the same side of the neutral value. However, it could be argued that for some experts, the presence of such score pairs could nevertheless contribute to opinion polarization. If this would be the case, possibly the associated frequencies could lead to better estimates of the weights. To test this, we set up an Opinion Polarization Index as


(4) $$\text{P}={\text{b}}_{0}\,{\text{f}}_{0}+{\text{b}}_{1}\,{\text{f}}_{1}+{\text{b}}_{2\,a}\,{\text{f}}_{2\,a}+{\text{b}}_{2\,b}\,{\text{f}}_{2\,b}+{\text{b}}_{3}\,{\text{f}}_{3}+{\text{b}}_{4}\,{\text{f}}_{4}$$

and again required its values to be between 0 and 100. It can be derived (see Supplementary Material Section 1.5.3) that because of this maximum and minimum value, our general expression reduces to


(5) $$\text{P}={\text{b}}_{1}\,{\text{f}}_{1}+{\text{b}}_{2\,a}\,{\text{f}}_{2\,a}+{\text{b}}_{2\,b}\,{\text{f}}_{2\,b}+{\text{b}}_{3}\,{\text{f}}_{3}+1.98\,{\text{f}}_{4}$$

Again, to ensure that *P* will not be negative, all weights will be required to be nonnegative and a minimal requirement to avoid that *P* exceeds 100 is that the weights should not exceed 1.98 (see also Supplementary Material Section 1.5.3).

For each of the 15 distributions, we now model the average expert judgments (*P*_*obs*_) as *P*_*obs*_ = *b*_1_*f*_1_ *+ b*_2_*_*a*_f*_2_*_*a*_* + *b*_2_*_*b*_f*_2_*_*b*_* + *b*_3_*f*_3_ + 1.98 × *f*_4_ + *e*, and we search the weights *b*_1_, *b*_2_*_*a*_, b*_2_*_*b*,_* and *b*_3_ such that we minimize the sum of squared error terms over the 15 distributions with the constraint that all weights are nonnegative, using the nonnegative least squares procedure by Lawson and Hanson (1974). The resulting weights were *b*_1_ = 0, *b*_2_*_*a*_* = 0, *b*_2_*_*b*_* = 1.07, and *b*_3_ = 1.35. In other words, implying the constraint that weights cannot be negative, led to zero weights for *f*_1_ and *f*_2_*_*a*_*, and thereby brought us automatically back to the same model as fitted above: *P*_*obs*_ = *b*_2_*f*_2_*_*b*_* + *b*_3_*f*_3_ + 1.98 × *f*_4_ + *e*, and obviously, the resulting estimates are the same as in Model (3). Thus, on the basis of these data, there is no reason to take into account frequencies of pairs of scores that are on the same side of the neutral value.

#### Comparison With Other Polarization Indices

To test how well the Opinion Polarization Index would predict expert judgments of polarization in comparison to existing polarization indices, we also predicted the expert judgments in our study with three commonly used polarization indices: Van der Eijk’s measure of polarization, the normed standard deviation, and the Esteban-Ray index and we compared the results.

##### Normalized SD

Different “standard” measures of dispersion have been used to assess polarization. The standard deviation may be the most common one. Before we compare this measure to our measures, we norm it such that it lies between 0 and 100, by dividing it by its maximal value (which for five-point scales is 2), and multiplying this by 100. Figure 2B, plots the normalized SD of the distributions against the mean expert ratings. From the scatter plot we see that for low to moderate values the normalized SD overestimates expert judgments, while for high values they are approximately equal. This lower fit (compared to our Opinion Polarization Index) is reflected in a higher RMSE = 27.92, 95% CI [25.2, 30.6].

##### Van der Eijk’s measure of polarization

Van Der Eijk (2001) proposed an alternative measure for polarization. Although initially developed to measure agreement in ordered rating scales, this measure was also translated into a measure of opinion polarization. The measure is a weighted average of the degree of agreement that exists in the simple component parts – layers – into which any frequency distribution can be disaggregated. Polarization scores range from 0 to 1. If all observations are in the same category, polarization is 0. With half the observations in one category, and half the observations in a different (non-neighboring) category, polarization is 1. Polarization is 0.5 for a uniform distribution over all categories. Using the *Agrmt*-Package in *R*, Van der Eijk’s polarization scores were calculated for each of the distributions in our study. Figure 2C, plots the scores according to Van der Eijk’s Polarization measure against the mean expert ratings. From the scatter plot we see that for low values Van der Eijk’s measure overestimates expert judgments, while for high values, Van der Eijk’s measure underestimates expert judgments. The vertical distances to the line are a bit higher, with the RMSE = 11.44, 95% CI = [9.6, 13.7] which means that, although better than the Normalized SD, the fit is appreciably worse than that of our Opinion Polarization Index.

##### Esteban and Ray’s index of polarization

Even though Esteban and Ray (1994) developed their measure to assess income polarization, their approach is similar to ours, in that it takes differences between pairs of scores within a distribution as a starting point for calculating polarization. Two important differences are observed that we believe make our Opinion Polarization Index more suitable for measuring opinion polarization: First, our index treats the midpoint as meaningful, and thus weighs pairs of scores differently depending on their location on the scale relative to the neutral opinion (see argument in the introduction). Second, our index is scaled from 0 to 100, which makes absolute scores on the index interpretable, whereas for the Esteban-Ray index, the absolute values change depending on the level of alpha. Esteban and Ray (1994, p. 834) write that alpha “may be treated as the degree of *polarization sensitivity* of the derived measure,” and they mention that its value should range between 0 and 1.6.

**FIGURE 3:**
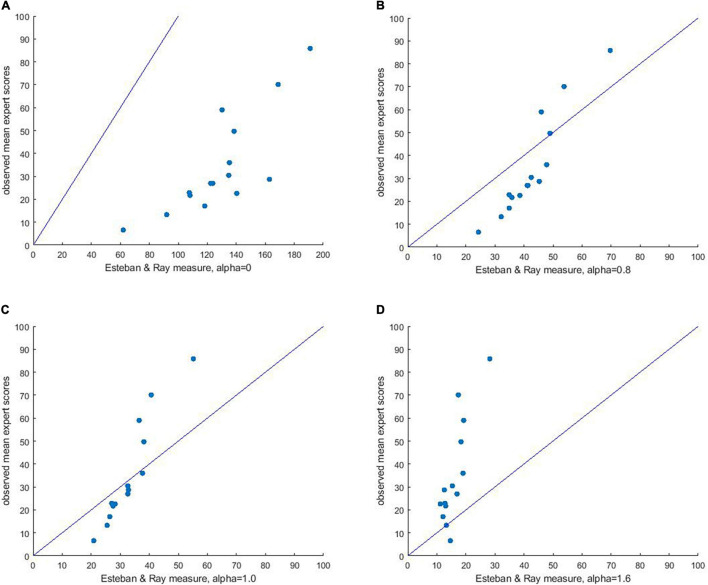
Plot of mean expert polarization scores for all 15 distributions against Esteban & Ray’s polarization index, with alpha = 0 **(A)**, 0.8 **(B)**, 1 **(C)**, and 1.6 **(D)**.

We used the R-package *acid* to compute the Esteban-Ray index (*polarization.ER*) for each of the 15 distributions of frequencies that we employed. We compared the predictive value of our Opinion Polarization Index to the predictions derived from the Esteban-Ray index at alpha = 0 (note that similar values are obtained when alpha is set to 0.01), alpha = 1.6 (their maximum level of alpha), and two intermediate levels: alpha = 0.8, and alpha = 1. Figures 3A–D, displays scatterplots predicting mean expert ratings at the *y*-axis, by the Esteban-Ray index at the *x*-axis.

A first observation is that a positive relation exists across alpha levels, but for the Esteban-Ray index, the absolute values strongly differ for the different levels of alpha. For instance, when alpha is set at 0, polarization scores of the provided distributions range from [61;190], whereas when alpha is set at 1.6, polarization scores range from [11;29]. This means that scores obtained from the Esteban-Ray index can only be compared to other scores at the same level of alpha, but have no absolute meaning. In contrast, scores on the polarization index range from [0;100], with 0 indicating no polarization, and 100 being the maximum level of polarization a distribution can take. Although, in principle, the index could be normalized and made comparable across different levels of alpha, this is not how Esteban and Ray developed the measure, and also not a function of the R-package *acid* which we used to calculate the Esteban-Ray index. Please note that Esteban and Ray did describe a scaling constant K, but this was used for a different kind of scaling (i.e., to take into account actual frequencies rather than proportions, 1994; p.849, section 5.1).

Because of the Esteban-Ray index consistently overestimates (when alpha = 0, Figure 3A), or underestimates (when alpha = 1.6, Figure 3D) the expert scores, the RMSE shows poor fit for both the minimum level of alpha (i.e., 0, RMSE = 96.22, 95% CI = [93.7, 98.8]), and the maximum level of alpha (i.e., 1.6, RMSE = 25.86, 95% CI = [24.3, 27.6]). When alpha is set to intermediate levels, vertical distances between the Esteban-Ray scores and the [0,100] line decrease. However, the scatter plots (Figures 3B,C) suggest that low expert polarization scores tend to be overestimated by the Esteban-Ray index, whereas high expert polarization scores tend to be underestimated. Moreover, while the fit improves for alpha = 0.8 (RMSE = 14.75, 95% CI = [12.5, 17.2]) and for alpha = 1 (RMSE = 14.30, 95% CI = [12.9, 16.0]), it is still poorer than the fit obtained with our Opinion Polarization Index.

#### Study of Possible Overfitting: Cross Validation Across Distributions

Because the predictive performance is assessed for the very same distributions as for which we optimized the weights for our Opinion Polarization index, the predictive performance may be overestimated. In other words, the weights may work quite well for the distributions we used, but might work less for other distributions, in which case we would suffer from overfitting. Therefore, it would be more appropriate to see how well the weights in our Opinion Polarization Index would predict distributions that were not in our sample. To this end, we decided to check the predictive validity of the Opinion Polarization Index by predicting the scores for each distribution, based on regression weights derived from our sample of distributions *excluding* the scores for that specific distribution. This so-called leave-one-out analysis allowed us to determine how well the Opinion Polarization Index based on *optimally estimated* weights predicts out-of-sample distribution scores, and hence is an indication for how well our optimally designed Opinion Polarization Index would actually work for unobserved distributions.

To this end, we calculated the optimal prediction weights based on 14 distributions, to predict the scores of the 15th distribution. Each time, a different distribution was left out. The regression weights for these 15 regressions usually differed very little from the ones obtained for the full set (*b*_*2*_=1.07 and *b*_3_=1.35), and their means were 1.05 and 1.35, respectively. There were some exceptions, the most extreme being the weights when leaving out Distribution 8 (*b*_2_=0.63 and *b*_3_=1.57) and Distribution 9 (*b*_2_=0.95 and *b*_3_=1.58). Other than this, *b*_*2*_ varied between 1.06 and 1.24, while *b*_*3*_ varied between 1.13 and 1.40.

We then computed the differences between predicted and mean scores for each left out distribution. Next, we computed the square root of the sum of squares of these differences, the RMSE, which is comparable to the RMSE values computed above. The resulting value was RSME = 9.56 (compared to RSME = 7.57 for in sample predictions). Compared to other measures, cross validation shows that our Opinion Polarization Index predicts out of sample distributions still better than those for Van der Eijk’s measure (RSME = 11.44) and the Normalized SD (RSME = 27.92), or Esteban and Ray’s index at alpha = 1 (RMSE = 14.30).

## Discussion

We believe the present research makes two important contributions: First, it provides a novel and valid measure for opinion polarization, and second, it presents a new methodological approach to develop measurements that are well-founded across disciplines: through “crowdsourcing” of expertise.

With increasing polarization in society rises the quest for measures to assess opinion polarization, in order to predict when conflict between societal groups may occur. Because of the widespread interest in opinion polarization across different fields, existing efforts to measure opinion polarization have often been informed by field-specific theoretical approaches, and therefore tend to focus on a specific characteristic of opinion polarization. The current paper aimed to integrate this cross-disciplinary knowledge by taking a novel methodology of measurement development. Rather than selecting a single characteristic of a distribution to assess opinion polarization (e.g., bimodality, dispersion), we integrate the judgments of polarization experts from political science, psychology, sociology, international relations, communication science, and economics to arrive at a valid opinion polarization measure.

This integrative approach resulted in the development of a novel Opinion Polarization Index. The index provides weights for pairs of individuals in an opinion distribution, in this case, over a 5-point scale. The weights of the index were derived from regression analysis on the expert scores. Importantly, the index takes into account many different aspects of a distribution that experts deem important contributors to polarization. Specifically, our qualitative analyses of the experts’ descriptions of polarization revealed four key characteristics of polarized opinion distributions: clustering, extremity, distance, and, to a somewhat lesser extent, balance. The most important aspect, mentioned by 77% of the experts, was the *clustering* of opinions into a (most often) bimodal distribution. Another important aspect was the *extremity* of opinions, or, in other words, the extent to which opinion differences were on opposing sides of the distribution (rather than different from the midpoint). The Opinion Polarization Index captures a combination of bimodality and extremity in assessing opinion polarization, because it captures the notion that opinions are clustered on two opposing sides of the underlying opinion spectrum. We showed that our best fitting model weighted only those pairings that fell on opposing sides of the neutral point on the underlying opinion spectrum. In fact, we found that including weights for difference pairs on either side of the distribution (e.g., pairs of 1–3, or 4–5) did not improve the predictive value of the index.

A third aspect, *distance* between opinions, is also incorporated in the Opinion Polarization Index, as 4-point distances between pairs of scores are weighed more heavily than 3-point distances, which, in turn, receive a higher weight than 2-point distances. This confirmed our expectation that opinion polarization mainly occurs when opinions are perceived as incompatible, because they are on opposite sides of the scale. Finally, the Opinion Polarization Index was sensitive to a *balance* of opinions, such that a more equal distribution of scores on each side of the distribution would result in higher Opinion Polarization Index scores.

Embodying all these aspects of opinion polarization, our Opinion Polarization Index proved to be a valid predictor of expert judgments of new opinion distributions that use the same 5-point scale. Importantly, it predicted the expert judgments better than existing measures of polarization, such as the standard deviation, the Esteban-Ray index, or Van der Eijk’s measure of polarization.

The Opinion Polarization Index should be suitable for measuring both *actual* opinion polarization (e.g., by examining a distribution of many individuals’ opinions on a topic obtained by a poll) and individual *perceptions* of opinion polarization (e.g., by asking a single individual how he/she thinks opinions on a certain topic are distributed in society). Previous research that aimed to assess perceived variability among members of a group has asked participants to distribute population representatives to the different categories of an opinion distribution (Kashima and Kashima, 1993; Kusumi et al., 2017). They used this variability (assessed with the standard deviation), in combination with the perceived norm, to predict people’s communication behaviors. Based on similar distributions one could calculate the Opinion Polarization Index to assess the predictive value of opinion polarization perceptions for emotions, cognitions and behaviors regarding potentially polarized topics in society.

### Limitations and Future Research

An important limitation is that this research relies on opinion polarization as can be derived from a distribution of opinions. Our focus on single opinion distributions has the advantage that it provides a simple and straightforward index that can be easily applied to a large set of situations even when only limited information may be available (i.e., a distribution of opinions on a single issue). On the downside, this focus limits our assessment of opinion polarization to opinions on a single topic. With the Opinion Polarization Index, we can therefore not take into account the way an opinion is embedded in a network of opinions, and is therefore likely to represent a broader ideological polarization (DiMaggio et al., 1996; Converse, 2006), nor can we account for its significance for one’s identity (e.g., Blau, 1977; DiMaggio et al., 1996), or the emotions attached to these attitudes (Clifford, 2019; Iyengar et al., 2019).

We therefore distinguish opinion polarization, which we measure with our index, from affective polarization, which is conceptualized as the animosity between those belonging to different parties or groups (Iyengar et al., 2019). Our index could predict the likelihood of opinion clusters being perceived as groups or categories (cf. Turner and Hogg, 1987). If clusters of opinions are defined in terms of opinion-based groups, they are negatively predictive of communication tendencies and emotions (Koudenburg and Kashima, 2021). This is because when opinion differences are seen as structural differences that define group membership, that are essentialized and unlikely to change, and pose a relational threat between those with different opinions. We therefore think it is important to devote future research to examining the circumstances that turn categorized opinion clusters into essentialized groups or categories. Our index could help to identify the first step toward structural or affective polarization, by identifying societal topics on which people are likely to be categorized into groups on the basis of their opinions. Indeed, we would expect that increased opinion polarization scores on our index would predict increased likelihood for categorization into opinion-based groups. Furthermore, the Opinion Polarization Index could be used to assess determinants of perceived opinion polarization. Indeed, research suggests that perceptions of polarization and actual levels of polarization may, at times, diverge (e.g., Fiorina et al., 2005). To assess the determinants of these perceptions, researchers may use experimental manipulations to see whether affective political discourse, or other types of information may foster subjects’ beliefs that opinions in society are polarized.

Based on the experience gained by this empirical way of constructing an opinion polarization measure, future research can be devoted to variants of the Opinion Polarization Index involving other numbers of opinion scale values. Another interesting venue is to extend the empirical approach to the development of other hard to measure concepts. We present a method for integrating knowledge that is scattered across disciplines, by simply using this knowledge as input for a regression model to predict the concept of interest. We believe such an approach could be readily used for other concepts, which are (1) in essence weighted combinations of subscores for which the weights are hard to decide on, (2) studied through many different (theoretical) approaches, and therefore can optimally benefit from the integration of expertise. The process we followed may be developed into a general method for constructing a consensual index of contested social scientific constructs.

## Data Availability Statement

The qualitative and quantitative data presented in the study are included in the Supplementary Material.

## Ethics Statement

The studies involving human participants were reviewed and approved by Ethics Committee Psychology of the University of Groningen. The participants provided their written informed consent to participate in this study.

## Author Contributions

All authors contributed to the study design and the development of the measure. NK supervised the recruitment of the experts and wrote the first version of the manuscript. HK conducted the quantitative analyses. YK and NK coded the data and conducted the qualitative analyses. All authors critically reflected on the analyses and revised the manuscript, and all authors agree to be accountable for the content of the work.

## Conflict of Interest

The authors declare that the research was conducted in the absence of any commercial or financial relationships that could be construed as a potential conflict of interest.

## Publisher’s Note

All claims expressed in this article are solely those of the authors and do not necessarily represent those of their affiliated organizations, or those of the publisher, the editors and the reviewers. Any product that may be evaluated in this article, or claim that may be made by its manufacturer, is not guaranteed or endorsed by the publisher.

## Footnotes

1 Van der Eijk extended his conclusions to measures that are transformations of the standard deviation, such as Granberg and Holmberg’s coefficient of consensus.

2 Six respondents indicated that they did not study polarization (anymore). We retained their data for analysis because their open answers suggested that they had well-developed views on polarization and/or had studied the topic before, and had clear views on how to assess opinion polarization from the distributions.

3 Upon writing *p*_*i*_ (*i* = 1,*…*,5) for the percentages of scores, and using that, e.g., *f*_2_*_*b*_* = ((*p*_2_*p*_4_ + *p*_4_*p*_2_)/9900) × 100, we can write (3) as

$$P=1.07\times \left(p_{2}p_{4}+p_{4}p_{2}\right)/99+1.35\times \left(p_{1}p_{4}+p_{4}p_{1}+p_{2}p_{5}+p_{5}p_{2}\right)/99+1.98\times \left(p_{1}p_{5}+p_{5}p_{1}\right)/99$$

. Next, denoting the score frequencies by *n*_*i*_ (*i* = 1,*…*,5), and their sum by *n*, we have *p*_*i*_ = 100(*n*_*i*_/*n*) which yields *P* = (2.14 × *n*_2_*n*_4_ + 2.70 × (*n*_1_*n*_4_ + *n*_2_*n*_5_) + 3.96 × *n*_1_*n*_5_)/(0.0099*n*^2^).
