# Supplementary material for: A New Opinion Polarization Index Developed by Integrating Expert Judgments
Source: Front Psychol. 2021 Oct 13;12:738258. doi: 10.3389/fpsyg.2021.738258 (PMC8549827; doi:10.3389/fpsyg.2021.738258)
Supplement: Supplementary file 3 [file Data_Sheet_3.docx]

Supplementary Material

# Step-by-Step Analyses Polarization Index

## Step 0. Obtaining scores on 15 plots from the actual experts.

Note that the raw data contain scores for four pilot subjects. These were ignored. The data for the remaining 60 experts were extracted, and saved in a Matlab file PolarizationDataRaw.doc

## Step 1. Data screening and Data correction

By checking minima and maxima we found a value 885 (for subject 22) which was an obvious error, and must have been a typo. Either 85 or 88 must have been meant. We chose 85, as participants mostly use values ending on 0 or 5. Indeed, 85 occurred often in the data, 88 never. Moreover, this choice instead of 88 will have hardly any effect on the outcomes. Note this, change was not saved in the Matlab file, but only in the script PolarizationAnalyses.m

% Checking understanding of the scale^[[1]](#footnote-1)^:

% We computed means per expert

mean(X,2)'

% we saw that mean for expert no.37 was very low. Scores turned out to be

% between 0 and 4; clearly this expert had not understood the scale.

X(37,:)

% 0 2 0 4 0 0 2 4 0 0 1 0 0 4 0

% We discarded this expert no 37.

X(37,:)=[];

% By checking minima and maxima of the remaining data, we found that scores ranged from at most 20 to at least 60, which seemed OK to us.

max(min(X'))

min(max(X'))

### Checking for consistency: Correlations between 59 experts

The minima in the correlation matrix were strongly negative. Scrutiny of the whole correlation matrix via a heatmap, see below, revealed that expert no. 29 showed negative correlations with all but one others, and most of them were quite strongly negative.

Fig S1. Heatmap of correlations between 59 experts (after exclusion expert no. 37).

Details for no.29

> R(29,:)

Columns 1 through 13

-0.2737 -0.2317 -0.6580 -0.3931 -0.4716 0.0936 -0.5271 -0.6237 -0.2618 -0.0755 -0.2175 -0.4150 -0.6108

Columns 14 through 26

-0.4137 -0.1500 -0.1568 -0.2645 -0.1135 -0.2698 -0.2073 -0.6167 -0.1649 -0.2273 -0.5890 -0.3697 -0.2320

Columns 27 through 39

-0.1685 -0.2998 1.0000 -0.5503 -0.2946 -0.4286 -0.4412 0.2398 -0.3584 -0.5911 -0.3066 -0.4383 -0.5786

Columns 40 through 52

-0.2676 -0.4422 -0.4039 -0.0113 -0.5097 -0.4863 -0.4123 -0.1437 -0.5199 -0.0637 -0.3636 -0.2441 -0.5846

Columns 53 through 60

-0.2378 -0.5636 -0.2493 -0.2742 -0.5899 -0.3247 -0.3896 -0.1871

Comparing data of no.29 with its neighbours clearly reveals a misunderstanding of the measurement scale (eg see 3^rd^ and 14^th^ column)

ans =

no.29 30 20 85 30 60 60 20 20 0 40 15 35 10 25 35

no.30 20 60 10 80 30 30 70 80 60 10 50 50 30 90 20

no.31 0 60 0 80 0 0 70 80 0 0 0 10 0 100 0

no.32 20 50 5 80 15 15 60 70 30 10 17 15 25 90 13

We therefore chose to also delete expert no.29 from the data.

Fig S2. Heatmap of correlations between 58 experts (after exclusion expert no. 29 and 37).

## Full data (after data cleaning).

**Table S1.** Full data, after data cleaning (see Step 1 and 2).

| **Expert** | **D1** | **D2** | **D3** | **D4** | **D5** | **D6** | **D7** | **D8** | **D9** | **D10** | **D11** | **D12** | **D13** | **D14** | **D15** |
| --- | --- | --- | --- | --- | --- | --- | --- | --- | --- | --- | --- | --- | --- | --- | --- |
| **1** | 0 | 60 | 0 | 80 | 10 | 10 | 30 | 70 | 0 | 0 | 30 | 10 | 0 | 95 | 10 |
| **2** | 15 | 25 | 0 | 60 | 25 | 25 | 20 | 75 | 0 | 10 | 25 | 25 | 30 | 90 | 25 |
| **3** | 40 | 60 | 20 | 70 | 45 | 45 | 60 | 70 | 80 | 35 | 50 | 35 | 50 | 90 | 40 |
| **4** | 30 | 40 | 10 | 60 | 50 | 50 | 50 | 50 | 50 | 30 | 40 | 35 | 40 | 80 | 60 |
| **5** | 10 | 20 | 5 | 75 | 5 | 5 | 30 | 60 | 40 | 5 | 20 | 30 | 30 | 85 | 40 |
| **6** | 10 | 30 | 15 | 80 | 80 | 80 | 75 | 80 | 0 | 0 | 70 | 30 | 0 | 90 | 30 |
| **7** | 20 | 30 | 10 | 80 | 20 | 20 | 80 | 80 | 50 | 15 | 35 | 25 | 40 | 90 | 25 |
| **8** | 50 | 60 | 10 | 80 | 20 | 20 | 70 | 70 | 50 | 20 | 40 | 30 | 40 | 90 | 30 |
| **9** | 0 | 20 | 0 | 90 | 0 | 0 | 20 | 75 | 10 | 0 | 10 | 0 | 0 | 100 | 0 |
| **10** | 0 | 20 | 0 | 80 | 50 | 50 | 80 | 80 | 0 | 0 | 40 | 40 | 0 | 90 | 40 |
| **11** | 3 | 10 | 1 | 50 | 0 | 0 | 10 | 70 | 0 | 5 | 5 | 0 | 2 | 90 | 0 |
| **12** | 25 | 70 | 5 | 95 | 20 | 20 | 65 | 90 | 15 | 25 | 40 | 25 | 30 | 85 | 25 |
| **13** | 25 | 45 | 15 | 80 | 30 | 35 | 55 | 60 | 75 | 20 | 40 | 25 | 50 | 85 | 30 |
| **14** | 25 | 25 | 5 | 50 | 15 | 15 | 32 | 30 | 20 | 15 | 20 | 20 | 30 | 75 | 20 |
| **15** | 25 | 40 | 10 | 70 | 60 | 60 | 50 | 40 | 30 | 20 | 45 | 35 | 40 | 80 | 33 |
| **16** | 10 | 20 | 0 | 30 | 20 | 20 | 20 | 20 | 5 | 10 | 15 | 10 | 10 | 60 | 15 |
| **17** | 20 | 60 | 10 | 85 | 50 | 52 | 80 | 75 | 30 | 15 | 40 | 35 | 25 | 90 | 35 |
| **18** | 20 | 30 | 19 | 80 | 30 | 30 | 40 | 40 | 20 | 10 | 20 | 30 | 20 | 80 | 30 |
| **19** | 10 | 10 | 5 | 50 | 10 | 10 | 40 | 30 | 20 | 5 | 10 | 10 | 10 | 90 | 10 |
| **20** | 1 | 5 | 0 | 70 | 10 | 10 | 55 | 55 | 1 | 0 | 10 | 0 | 1 | 80 | 1 |
| **21** | 20 | 35 | 5 | 70 | 12 | 12 | 60 | 57 | 50 | 15 | 30 | 17 | 40 | 85 | 20 |
| **22** | 20 | 60 | 10 | 80 | 65 | 65 | 70 | 60 | 20 | 15 | 60 | 25 | 35 | 85 | 25 |
| **23** | 0 | 50 | 0 | 80 | 20 | 20 | 60 | 70 | 0 | 0 | 20 | 10 | 0 | 90 | 10 |
| **24** | 15 | 42 | 5 | 45 | 15 | 15 | 32 | 20 | 40 | 10 | 40 | 12 | 30 | 70 | 12 |
| **25** | 15 | 38 | 6 | 80 | 30 | 30 | 49 | 61 | 40 | 11 | 32 | 35 | 20 | 95 | 25 |
| **26** | 0 | 30 | 0 | 75 | 15 | 15 | 60 | 80 | 0 | 0 | 15 | 10 | 0 | 85 | 10 |
| **27** | 0 | 0 | 0 | 75 | 0 | 0 | 10 | 50 | 0 | 0 | 0 | 0 | 0 | 90 | 0 |
| **28** | 0 | 50 | 0 | 70 | 0 | 0 | 60 | 40 | 0 | 0 | 10 | 0 | 0 | 80 | 0 |
| **29** | 20 | 60 | 10 | 80 | 30 | 30 | 70 | 80 | 60 | 10 | 50 | 50 | 30 | 90 | 20 |
| **30** | 0 | 60 | 0 | 80 | 0 | 0 | 70 | 80 | 0 | 0 | 0 | 10 | 0 | 100 | 0 |
| **31** | 20 | 50 | 5 | 80 | 15 | 15 | 60 | 70 | 30 | 10 | 17 | 15 | 25 | 90 | 13 |
| **32** | 40 | 55 | 15 | 70 | 63 | 67 | 65 | 60 | 60 | 35 | 60 | 35 | 50 | 80 | 40 |
| **33** | 0 | 20 | 0 | 60 | 80 | 80 | 45 | 20 | 0 | 0 | 10 | 10 | 0 | 90 | 10 |
| **34** | 10 | 30 | 5 | 70 | 30 | 30 | 30 | 40 | 50 | 20 | 30 | 30 | 20 | 90 | 40 |
| **35** | 25 | 40 | 10 | 65 | 15 | 15 | 60 | 70 | 65 | 60 | 40 | 40 | 25 | 90 | 40 |
| **36** | 60 | 40 | 10 | 80 | 25 | 25 | 35 | 75 | 25 | 35 | 30 | 40 | 55 | 80 | 30 |
| **37** | 20 | 60 | 5 | 70 | 15 | 15 | 60 | 80 | 50 | 20 | 50 | 18 | 20 | 90 | 15 |
| **38** | 15 | 40 | 10 | 80 | 35 | 35 | 65 | 70 | 25 | 10 | 35 | 20 | 15 | 85 | 20 |
| **39** | 30 | 40 | 10 | 45 | 30 | 30 | 40 | 55 | 30 | 20 | 30 | 35 | 35 | 80 | 40 |
| **40** | 5 | 40 | 0 | 55 | 30 | 30 | 50 | 55 | 40 | 15 | 40 | 20 | 10 | 90 | 20 |
| **41** | 50 | 40 | 30 | 60 | 40 | 40 | 50 | 60 | 0 | 50 | 40 | 50 | 50 | 80 | 50 |
| **42** | 25 | 50 | 10 | 60 | 35 | 35 | 45 | 50 | 50 | 15 | 50 | 20 | 35 | 90 | 20 |
| **43** | 20 | 15 | 10 | 70 | 9 | 9 | 60 | 40 | 50 | 15 | 20 | 20 | 30 | 80 | 50 |
| **44** | 10 | 20 | 0 | 80 | 15 | 15 | 50 | 45 | 40 | 5 | 20 | 10 | 20 | 90 | 5 |
| **45** | 10 | 50 | 5 | 80 | 40 | 40 | 50 | 70 | 5 | 0 | 30 | 25 | 10 | 90 | 30 |
| **46** | 15 | 30 | 5 | 80 | 30 | 30 | 60 | 70 | 70 | 10 | 60 | 50 | 20 | 95 | 50 |
| **47** | 0 | 20 | 3 | 50 | 33 | 33 | 33 | 50 | 6 | 0 | 22 | 18 | 1 | 70 | 20 |
| **48** | 0 | 15 | 5 | 100 | 15 | 15 | 20 | 90 | 90 | 0 | 10 | 5 | 0 | 95 | 5 |
| **49** | 10 | 10 | 10 | 90 | 10 | 10 | 90 | 90 | 10 | 10 | 10 | 10 | 10 | 90 | 10 |
| **50** | 20 | 30 | 5 | 60 | 10 | 10 | 30 | 40 | 40 | 15 | 30 | 20 | 30 | 75 | 20 |
| **51** | 0 | 30 | 0 | 75 | 5 | 5 | 50 | 75 | 0 | 0 | 0 | 5 | 0 | 100 | 0 |
| **52** | 20 | 35 | 5 | 48 | 33 | 33 | 40 | 47 | 48 | 10 | 40 | 15 | 38 | 70 | 15 |
| **53** | 20 | 20 | 5 | 40 | 20 | 20 | 40 | 40 | 15 | 10 | 10 | 10 | 20 | 90 | 15 |
| **54** | 30 | 60 | 10 | 80 | 40 | 40 | 65 | 70 | 3 | 20 | 50 | 39 | 40 | 90 | 39 |
| **55** | 50 | 25 | 10 | 75 | 30 | 30 | 45 | 30 | 60 | 25 | 45 | 35 | 65 | 85 | 45 |
| **56** | 10 | 40 | 0 | 70 | 10 | 10 | 30 | 80 | 10 | 5 | 25 | 5 | 10 | 100 | 5 |
| **57** | 30 | 57 | 20 | 70 | 58 | 58 | 65 | 45 | 50 | 35 | 58 | 36 | 50 | 75 | 40 |
| **58** | 10 | 15 | 5 | 50 | 50 | 50 | 40 | 20 | 40 | 10 | 45 | 10 | 30 | 70 | 20 |

## Step 2. Exploratory analysis

2D MDS on Euclidean Distances between 58 rows (using an mds program obtained from Groenen)

The idea was to see if there were clear clusters in response patterns. We only tried 2-dim., and it turned out that this gave a very good fit. We saw no clear clustering

>> [A,s,fitperc]=mds(DIST(Data),2,0,1e-8,1)

Stress after 98 iters = 0.04455793 0.00000001

A =

-51.4139 -31.7687

-30.5930 -5.8338

83.3033 24.4305

62.5590 22.1256

-19.7067 32.6557

46.9337 -93.2718

26.6991 -12.5156

45.8841 -5.3105

-82.3553 -7.3208

8.6938 -73.4572

-87.9535 15.3042

17.1269 -48.6534

49.3853 26.1520

-26.5159 40.0502

56.3776 5.4958

-60.7185 52.9349

44.5361 -40.2553

-8.1118 9.5526

-53.5810 30.2414

-69.1530 -2.8928

5.8548 17.0577

57.4611 -47.9735

-35.7014 -38.1343

-15.6170 57.0907

9.4826 -3.3390

-44.8632 -34.6320

-93.2907 17.8033

-72.2421 -21.0090

49.7241 -20.9579

-65.4127 -51.7712

-11.7232 -16.4652

92.2005 2.9707

-15.0203 -102.2054

7.8446 32.4566

44.4217 54.9234

48.6499 41.9290

17.4807 -19.2499

4.0633 -23.2656

20.8192 23.9508

-0.5243 1.2175

80.8586 -28.2380

28.8659 14.8573

1.2004 51.1009

-28.3064 13.6766

-4.2997 -38.8155

62.5174 -3.7686

-46.0917 10.4728

-43.7057 91.2216

-41.6931 -61.3582

-12.7591 36.9366

-67.6831 -28.3359

13.2717 36.7899

-40.4443 27.7952

38.5996 -42.7772

58.4894 56.9484

-47.2909 -16.1501

82.5145 4.6214

10.9523 66.9632

s =

0.0446

fitperc =

95.5442

**Fig S3.** 2-dimensional MDS on Euclidean Distances between 58 rows.

## Step 3. Setting up the predictor matrix

The distributions were offered in the form of histograms. These were originally generated manually and chosen to have total frequencies equal to 100, but due to inadvertent truncations in the plots, this is not always the case. In distribution 3, the highest plotted frequency was 44, while 80 was intended, and in distributions 5,6 and 10, the highest plotted frequency was 44, while 45 was intended. We hence used the actually plotted frequencies in our further analyses.

The 15 rows of frequencies below correspond to the histograms presented to the experts (see Fig 1).

12 20 40 21 7

40 11 28 19 2

2 8 44 9 1

20 25 2 28 25

3 11 17 24 44

44 25 16 10 4

2 31 8 29 30

7 38 8 37 10

21 19 19 20 21

3 25 44 24 3

36 20 27 11 6

1 30 37 21 11

15 21 30 20 14

36 10 8 11 35

10 21 38 29 2

To make these rows comparable, we divided them by their sums, and multiplied them by 100, so that all rows added up to 100 and hence contain (rowwise) percentages of scores:

| 12.0000 | 20.0000 | 40.0000 | 21.0000 | 7.0000 |
| --- | --- | --- | --- | --- |
| 40.0000 | 11.0000 | 28.0000 | 19.0000 | 2.0000 |
| 3.1250 | 12.5000 | 68.7500 | 14.0625 | 1.5625 |
| 20.0000 | 25.0000 | 2.0000 | 28.0000 | 25.0000 |
| 3.0303 | 11.1111 | 17.1717 | 24.2424 | 44.4444 |
| 44.4444 | 25.2525 | 16.1616 | 10.1010 | 4.0404 |
| 2.0000 | 31.0000 | 8.0000 | 29.0000 | 30.0000 |
| 7.0000 | 38.0000 | 8.0000 | 37.0000 | 10.0000 |
| 21.0000 | 19.0000 | 19.0000 | 20.0000 | 21.0000 |
| 3.0303 | 25.2525 | 44.4444 | 24.2424 | 3.0303 |
| 36.0000 | 20.0000 | 27.0000 | 11.0000 | 6.0000 |
| 1.0000 | 30.0000 | 37.0000 | 21.0000 | 11.0000 |
| 15.0000 | 21.0000 | 30.0000 | 20.0000 | 14.0000 |
| 36.0000 | 10.0000 | 8.0000 | 11.0000 | 35.0000 |
| 10.0000 | 21.0000 | 38.0000 | 29.0000 | 2.0000 |

Now, in order to set up our ‘determinants’ of polarization, for each distribution, we computed how often different types of score pairs occurred (note these numbers are based on percentages of scores, so they need not be natural numbers):

f0=-n;for i=1:5,f0=f0+Pairs(i,i);end;
% Note: -n is to correct for pairs of indiv with itself

f1=0;for i=1:4,f1=f1+Pairs(i,i+1);end;f1=2*f1;

f2a=2*(Pairs(1,3)+Pairs(3,5));

f2s=2*Pairs(2,4);

f3=2*(Pairs(1,4)+Pairs(2,5));

f4=2*Pairs(1,5);

f0= # pairs of equal scores

f1= # pairs of scores differing 1 point

f2a= # pairs of scores differing 2 points on same side (i.e., (1,3) and (3,5))

f2b= # pairs of scores differing 2 points on different sides (i.e., (2,4))

f3= # pairs of scores differing 3 points

f4= # pairs of scores differing 4 points

Note, score pairs (i,j) and (j,i) have both been counted, even though obviously they are equivalent.

Score pairs (i,i) i.e. of an individual with itself have been discarded (in total there were by definition 100 such score pairs, because each profile related to 100 scores).

After counting the score pairs, the counts were normalized by dividing by the total number of pairs (=10000-100=9900 in all cases, because all profiles added up to 100) and by multiplying by 100, just to make them palatable numbers, which add up row wise to 100.

These scores form 6 possible characteristics of polarization. These scores actually are *percentages* of comparisons that fall in each of the six categories defined. And our study tries to assess how these should be weighted to get a good polarization measure.

The scores, rounded to four decimals, are given below:

**Table S2.** Percentage of score pairs in each category, for each of the distributions.

| **Distrib. no.** | **f0** | **f1** | **f2a** | **f2b** | **f3** | **f4** |
| --- | --- | --- | --- | --- | --- | --- |
| 1 | 25.5960 | 40.9495 | 15.3535 | 8.4848 | 7.9192 | 1.6970 |
| 2 | 27.9798 | 26.6263 | 23.7576 | 4.2222 | 15.7980 | 1.6162 |
| 3 | 50.4321 | 38.1254 | 6.5104 | 3.5511 | 1.2824 | 0.0986 |
| 4 | 23.6162 | 26.3838 | 1.8182 | 14.1414 | 23.9394 | 10.1010 |
| 5 | 29.1971 | 34.7109 | 16.4692 | 5.4416 | 11.4604 | 2.7208 |
| 6 | 29.2177 | 35.0407 | 15.8302 | 5.1531 | 11.1306 | 3.6277 |
| 7 | 26.9697 | 28.5253 | 5.1717 | 18.1616 | 19.9596 | 1.2121 |
| 8 | 29.5556 | 24.9697 | 2.7475 | 28.4040 | 12.9091 | 1.4141 |
| 9 | 19.2323 | 31.5152 | 16.1212 | 7.6768 | 16.5455 | 8.9091 |
| 10 | 31.5056 | 47.4699 | 5.4416 | 12.3673 | 3.0300 | 0.1855 |
| 11 | 25.0707 | 32.7879 | 22.9091 | 4.4444 | 10.4242 | 4.3636 |
| 12 | 27.5960 | 43.3939 | 8.9697 | 12.7273 | 7.0909 | 0.2222 |
| 13 | 20.8283 | 36.8687 | 17.5758 | 8.4848 | 12.0000 | 4.2424 |
| 14 | 27.3333 | 18.4444 | 11.4747 | 2.2222 | 15.0707 | 25.4545 |
| 15 | 27.5758 | 43.7980 | 9.2121 | 12.3030 | 6.7071 | 0.4040 |

It has been tried to set up the distributions such that they vary widely in characteristics, and such that the characteristics themselves have substantively different contributions. To see to what extent this was successful, we inspected the correlation matrix (Table 1 in manuscript).

Clearly, there are some strong correlations that could destabilize regressions. However, the characteristics affecting degree of polarization can be limited to those relating to where we have pairs of scores on both sides of the neutral scale value, considering that polarization that are those where idea is to define polarization on the basis of character

### Estimating the Polarization Index weights

Consider that only f2b, f3 and f4 contribute to polarization (because these count pairs of scores on either side of neutral). We wish to devise a measure of polarization according to

1. P=b2*f2b+b3*f3+b4*f4 and maximum 100 and minimum 0

*P* = 100 is the maximum, and it is obtained if the percentages of scores for both 1 and 5 are 50%. For convenience, we here consider that we actually have 100 observations, which implies that percentages of scores can be treated as frequencies of scores. Now the requirement is that *P*=100 if there are 50 scores of 1 and 50 scores of 5. In this distribution, pairs of differences of 2 or 3 points do not occur, hence *f*_2b_=0 and *f*_3_=0. Furthermore, *f_4_* equals the numbers of pairs (1,5) and (5,1), which is 50×50×2, divided by the total number of pairs, which is 100×100-100=9900, hence *f*_4_=(5000/9900)×100=5000/99= 50.51. So we have that, if there are 50 scores of 1 and 50 scores of 5, the expression for *P* becomes *P=b_2_f_2b_+b_3_f_3_+b_4_f_4_ =0+0+b_4_* ×50.51 = *b_4_* ×50.51. For this case we require that *P* equals 100, so we have *b_4_*×50.51 =100, hence *b_4_* =1.98. It follows that our general expression *P=b_2_f_2b_+b_3_f_3_+b_4_f_4_* can now be simplified into

1. P=b_2_f_2_b+b_3_f_3_+1.98f_4_

By fixing *b_4_* =1.98, we ensure that *P*=100 if the percentage of scores for both 1 and 5 is 50%. However, this does not yet ensure that 100 is the maximum attainable value for *P*. Indeed, if, for example, *b_3_* =3, we would get *P=* 151.53 if there were 50 scores of 1 and 50 scores of 4 (because then *f*_3_ would be 50.51). It can be shown^[[2]](#footnote-2)^, however, that if both *b*_2_ and *b*_3_ are smaller than or equal to *1.98*, *P* cannot exceed 100.

Furthermore, we require that *P* = 0 is the minimum, and that this is attained if all scores are on the same side of neutral (neutral included), hence when either all scores are 1, 2 or 3, or all scores 3, 4 or 5. Indeed, if all scores are on the same side of neutral, *f_2b_*=0, *f_3_*=0 and *f_4_*=0, hence indeed *P*=0. This does not yet imply that 0 is the minimal value of *P*. Indeed, if, for example, *b_3_* would be negative, then if there were 50 scores of 1 and 50 scores of 4, *P* would be a negative value. To avoid this, we must ensure that the weights *b_2_* and *b_3_* cannot be negative.

Thus we have only two free parameters left, b2 and b3.

We now wish to estimate these by fitting || y - (b2*f2b + b3*f3 + 1.98*f4) ||

where y contains expert Polarisation scores on a scale of 0 to 100

For this we did a simple intercept free regression of y-1.98*f4 on the two predictors f2b and f3

So we minimize || (y-1.98*f4)- (b2*f2b + b3*f3) ||

We did regression on the mean polarization scores across experts. (It is easy to prove that this gives the same results as taking means of regression weights for all experts).

**Table S3.** Mean expert scores per distribution.

| **Distrib. no.** | Mean expert score (y_mean) |
| --- | --- |
| 1 | 16.9655 |
| 2 | 35.8966 |
| 3 | 6.6207 |
| 4 | 70.0517 |
| 5 | 26.8621 |
| 6 | 27.0517 |
| 7 | 49.5862 |
| 8 | 59.0517 |
| 9 | 28.7586 |
| 10 | 13.1207 |
| 11 | 30.5000 |
| 12 | 21.8103 |
| 13 | 22.7069 |
| 14 | 85.9483 |
| 15 | 22.8966 |

Regression weights b2 and b3^^[[3]](#footnote-3)^^:

b =

1.0655

1.3480

With bootstrap Cis^^[[4]](#footnote-4)^^

for b2: [ 0.93, 1.20] and for b3: [1.19 , 1.51]

Using this, the resulting formula for P would be

P = 1.07 x f2b + 1.35 x f3 + 1.98 f4.

or more explicitly:

P = 1.07 x % of score pairs (2,4) + 1.35 x % of score pairs (1,4),(2,5) + 1.98 x % of score pairs (1,5)

### Model fit

As a fit measure, we computed the proportion unexplained sums of squares

proportion unexplained sum of squares = 0.0348

Bootstrap 95%CI =[ 0.022 , 0.056]

So, this gives a 96.5% fit. A disadvantage is that such a fit measure tends to be high. Therefore, it is better to focus on the Root mean squared error of estimation (RMSEA)

Root mean squared error of estimation = 7.5742

Bootstrap 95% CI= [ 6.11, 9.49]

It is easily interpreted, as it means that a ‘representative’ difference is 7.57

(It resembles the mean absolute difference, but weights big differences more heavily, like we do in our optimization in regression).

These measures are not well known and a bit unconventional, since usually proportion unexplained variance is used, but using variance here implies that we do not care about offset differences (e.g. one prediction systematically higher or lower than the expert scores), and actually, we do care. (Just for the record, we did compute

ssq(c(y_mean-1.98*X(:,6)-Xp*b))/ssq(c(y_mean)) % proportion unexplained variance

ans =

0.1245 So percentage explained variance in y is 87.5%)

### Including pairs with scores on the same side of the distribution

Above we considered that only f2b, f3 and f4 contribute to polarization (because these count pairs of scores on either side of neutral). Now let’s remove this assumption, and let’s assume that any of the frequencies can play a role in the prediction. We wish to devise a measure of polarization according to

1. P=b0*f0+ b1*f1+b2a*f2a+ b2b*f2b+b3*f3+b4*f4 and maximum 100 and minimum 0.^[[5]](#footnote-5)^

We again have the requirement that *P* = 100 is the maximum, and is attained if the percentage of scores for both 1 and 5 are 50%. If percentage of scores for both 1 and 5 are 50%, pairs of differences of 1, 2 or 3 points do not occur, hence *f_1_*, *f_2a_*, *f_2b_*, and *f_3_* are all 0. The frequency of pairs with equal scores is 4900 and that of pairs with score differences of 4 is 5000, see above. So we have *f_0_*=49.49 and *f_4_*=50.51, while the frequencies *f_1_*, *f_2a_*, *f_2b_*, and *f_3_* are all 0. So if the percentage of scores for both 1 and 5 are 50%, *P*= *b*_0_*f_0_*+*b*_4_*f_4_* = *b*_0_×49.49 + *b*_4_×50.51, which must equal 100, because it reflects maximal polarization.

Minimal polarization should be 0, and must be attained (at least^^[[6]](#footnote-6)^^) when all scores are equal, as is in line with the anchoring used in the experiment. So in this case *f_1_*=*f_2a_*=*f_2b_*=*f_3_*=*f_4_*=0, and *f_0_*=(9900/9900)×100=100. Clearly then *P*=*b*_0_×*f_0_*=*b*_0_×100 and this must equal 0, hence *b*_0_=0. Combining this with *b*_0_×49.49 + *b*_4_×50.51 = 100, we get *b*_4_×50.51 = 100, hence again *b*_4_= 1.98. So it follows that our general expression reduces to

P=b0f0+b_1_f_1_+b2af2a+b2bf2b+b_3_f_3_+b_4_f_4_

can now be simplified into

P=b_1_f_1_+b2af2a+b2bf2b+b_3_f_3_+1.98f_4_

Thus we now have four free parameters left, b1, b2a, b2b, and b3, while b0=0 and b4=1.98.

We now wish to estimate these by fitting || y - (b1*f1 + b2b*f2b + b2b*f2b + b3*f3 + 1.98*f4) ||

where y contains expert Polarization scores on a scale of 0 to 100

For this we did a simple intercept free regression of y-1.98*f4 on the four predictors f1, f2a, f2b and f3, so we minimize || (y-1.98*f4)- (b1*f1 + b2b*f2b + b2*f2b + b3*f3) ||

>> y_mean=mean(Data,1)' %y_mean contains averages over experts

Xp=X(:,2:5); % variables f1, f2a, fb and f3

b=Xp\(y_mean-1.98*X(:,6))

disp('proportion unexplained sum of squares')

ssq(y_mean-1.98*X(:,6)-Xp*b)/ssq(y_mean)

disp('Root mean squared error of estimation')

sqrt(ssq(y_mean-1.98*X(:,6)-Xp*b)/15)

y_mean =

16.9655

35.8966

6.6207

70.0517

26.8621

27.0517

49.5862

59.0517

28.7586

13.1207

30.5000

21.8103

22.7069

85.9483

22.8966

b =

-0.1609

0.0760

1.3021

1.4569

Bootstrap lower and upper bounds of Cis are

b_lo =

-0.2453 -0.0971 1.1041 1.2939

b_up =

-0.0725 0.2466 1.4916 1.6163

proportion unexplained sum of squares

ans =

0.0309

Bootstrap CI: [0.0208, 0.0454]

Root mean squared error of estimation

ans =

7.1410Bootstrap CI=[5.8551 , 8.6562]

However, a coefficient with a negative weight is unacceptable, because it can lead to negative Polarization values. So we have to enforce the constraint that all weights should be non-negative. To do so, we constrained that all weights are nonnegative, using the nonnegative least squares procedure by (Lawson & Hanson, 1974).

y_mean=mean(Data,1)' %y_mean contains averages over experts

Xp=X(:,2:5); % variables f1, f2a, fb and f3

b=NNLS(Xp,y_mean-1.98*X(:,6))

disp('proportion unexplained sum of squares')

ssq(y_mean-1.98*X(:,6)-Xp*b)/ssq(y_mean)

disp('Root mean squared error of estimation')

sqrt(ssq(y_mean-1.98*X(:,6)-Xp*b)/15)

The results were:

b =

0

0

1.0655

1.3480

proportion unexplained sum of squares

ans =

0.0348

Root mean squared error of estimation

ans =

7.5742

>>

So we can conclude that the necessary constraint that weights should be nonnegative actually leads to estimates for b1 and b2a that are 0. As a consequence, we actually fitted P = b2b*f2b + b3*f3 + 1.98*f4, and we get the very same results as in our first estimation where we used only f2b and f3 to start with.

## Comparison with other polarization indices

### Van der Eijk’s measure

Using Agrmt, and function Polarization, applied to vector of frequencies;

Help function says:

This function calculates polarization by re-scaling agreement A introduced by Cees van der Eijk. Whereas agreement A ranges from -1 to 1, polarization ranges from 0 to 1. If all observations are in the same category, polarization is 0. With half the observations in one category, and half the observations in a different (non-neighbouring) category, polarization is 1. Polarization is 0.5 for a uniform distribution over all categories.

I used Polarization(V), which actually does Polarization(V, old = FALSE)

**Arguments**

| V | A frequency vector |
| --- | --- |
| old | Specify old=TRUE to use the depreciated algorithm for agreement A |

PvdE =

0.3125

0.3667

0.1367

0.5267

0.2601

0.2601

0.4342

0.4417

0.5142

0.2374

0.3508

0.2725

0.4050

0.7447

0.2725

>> disp('Root mean squared error of estimation')

sqrt(ssq(y_mean-PvdE*100)/15)

Root mean squared error of estimation = 11.4367

95% CI = [9.6, 13.7]

(For the record: ssq(c(y_mean-PvdE*100))/ssq(c(y_mean)) % proportion unexplained variance

ans = 0.2727 So fit percentage is 77% )

### Normalized SD (standard deviation divided by maximum possible SD, which equals 2)

>> sd_norm'

ans =

0.5390

0.6160

0.3365

0.7604

0.5770

0.5857

0.6302

0.5974

0.7194

0.4293

0.6152

0.4944

0.6263

0.8732

0.4934

Root mean squared error of estimation

>> sqrt(ssq(y_mean-sd_norm'*100)/15) = 27.9160

95% CI =[25.2, 30.6]

(For the record: ssq(c(y_mean-sd_norm'*100))/ssq(c(y_mean)) % proportion unexplained variance

ans = 0.3616 So fit percentage is 64.8%)

### Esteban-Ray Polarization index

# package acid

pol=rep(-1,15)

POL=cbind(pol,pol,pol)

alpha=0.8 <<similar commands were defined for alpha = 0, 1, and 1.6 >>

means=c(1,2,3,4,5)

for distribution 1 <<similar commands were defined for all 15 distributions>> :

shares=c(12, 20, 40, 21, 7)

shares<-shares/sum(shares)

rho=data.frame(means=means,shares=shares)

p=polarisation.ER(alpha,rho,comp=FALSE)

pol[1]=p$P

**ER_A0 ER_A0.8 ER_A1 ER_A1.6**

117.98 34.89 26.44 12.15

135.08 47.89 37.73 19.02

61.87 24.35 20.94 14.70

168.82 53.77 40.57 17.50

122.56 41.30 32.62 17.00

123.66 41.16 32.50 16.98

138.52 48.87 38.13 18.33

130.34 46.02 36.58 19.13

162.74 45.27 32.88 12.61

91.99 32.23 25.53 13.15

134.86 42.51 32.64 15.35

107.86 35.76 27.64 13.12

140.54 38.59 28.16 11.16

190.94 69.67 55.24 28.30

107.48 35.01 27.05 12.89

sqrt(ssq(y_mean-ER_A1)/15)

sqrt(ssq(y_mean-ER_A1.6)/15)

sqrt(ssq(y_mean-ER_A0)/15)

sqrt(ssq(y_mean-ER_A0.8)/15)

## Study of possible overfitting: Cross-validation across distributions

% Verify overfitting effect:

% regression weights are optimized for the 15 distributions chosen.

% but to what extent are the regression weights too optimistic (i.e. overfitting).

% A leave one out cross validation study can give us some information on this. Do the regression weights differ for different distributions?

ss_LOOCV=[];BB=[];

y_mean=mean(Data,1)';

for i=1:15

% for data for 14 distribtutions (each time a different one left out) we

% compute prediction weights and assess how well they predict the scores

% for the left out distribution

y_meanii=y_mean;

y_meanii(i)=[]; % leave out mean scores for distr i

Xii=X;

Xii(i,:)=[]; % leave out predictors for distr i

Xp=Xii(:,4:5);

b=Xp\(y_meanii-1.98*Xii(:,6));
 BB=[BB b]; % next compute sum of squared distances between predicted and observed scores for expert i

% squared difference when prediction formula based on 14 other distribs is applied to distr i')

ss_LOOCV(i)=(y_mean(i)-1.98*X(i,6)-X(i,4:5)*b)^2;

end;

disp('root mean squared difference for OUT OF SAMPLE predictions');

sqrt(mean(ss_LOOCV))

% compare predictions based on full set of regr weights:

% ss holds squared difference for reg weights based on all 15 distrs

ss=(y_mean-1.98*X(:,6)-X(:,4:5)*b).^2;

disp('Check, recompute root mean squared difference for IN SAMPLE predictions');

sqrt(mean(ss))

% compare squared differences between y_mean and predicted value

[ss ss_LOOCV']

ans =

37.3694 40.0343

47.6622 67.8012

0.8304 0.8493

7.3536 12.5955

0.0520 0.0591

0.3918 0.4419

0.8539 1.2646

73.4877 330.0761

374.9798 494.5624

20.3842 27.1626

9.4415 10.5527

3.0790 3.7217

119.0614 133.6086

165.5816 247.6304

0.0035 0.0035

root mean squared difference for OUT OF SAMPLE predictions

ans =

9.5581

Check, recompute root mean squared difference for IN SAMPLE predictions

ans =

7.5742

Conclusion: Clearly, out of sample prediction sare a bit poorer, but, when comparing this Cross validated RMSE to those for vdEijk and Normed SD, regression predicts still better, see Table S4.

For the interest of the reader, we also report the different regression weights for the 15 regressions (in which one distribution was left out).

| **Left out distribution** | **b_2_** | **b_3_** |
| --- | --- | --- |
| 1 | 1.09 | 1.35 |
| 2 | 1.14 | 1.24 |
| 3 | 1.06 | 1.35 |
| 4 | 1.08 | 1.30 |
| 5 | 1.07 | 1.35 |
| 6 | 1.06 | 1.35 |
| 7 | 1.06 | 1.34 |
| 8 | 0.63 | 1.57 |
| 9 | 0.95 | 1.58 |
| 10 | 1.13 | 1.31 |
| 11 | 1.08 | 1.33 |
| 12 | 1.08 | 1.34 |
| 13 | 1.06 | 1.40 |
| 14 | 1.24 | 1.13 |
| 15 | 1.07 | 1.35 |
| Mean | 1.05 | 1.35 |
| SD | .13 | .11 |

Boxplot of the b2 and b3:


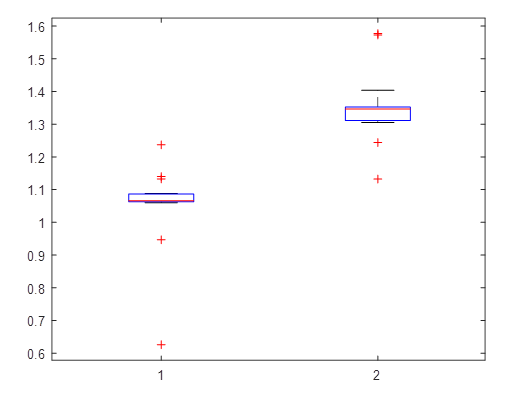


Given that we now see that leaving out Distribution 8 would lead to quite different regression weights, one may wonder whether leaving out 8 would actually be a good idea. We checked this by computing the associated RMSEA:

>> b=[0.625978  1.572606]'

disp('Root mean squared error of estimation')

sqrt(ssq(y_mean([1:7 9:15])-1.98*X([1:7 9:15],6)-X([1:7 9:15],4:5)*b)/14)

b =

    0.6260

    1.5726

Root mean squared error of estimation

ans =

    7.0940

It is quite a bit smaller than our one. However, it seems too opportunistic to leave Distr 8 out after having seen the data and the cross validation, so such a step would only be warranted if it could be tested on new data.

**1.7.1 compare different polarization indices including leave-one-out strategy**

Note that for vd Eijk’s measure and normed SD prediction formula does not depend on which distributions were in study.

Compare squared errors per distribution:

% compare squared diferences between y_mean and predicted value, regr in

% sample, reg out of sample, vdEijk, normed SD');

writescr([ss ss_LOOCV' ss_E ss_S],'4.2')

**Table S4.** Squared errors per distripution for the in-sample predictions, out-of sample predictions, vd Eijks measure of polarization, and the normed standard deviation.

| Distr no. | Regr in sample | Regr out sample | vdEijk | Normed SD |
| --- | --- | --- | --- | --- |
| 1 | 37.37 | 40.03 | 204.05 | 1363.84 |
| 2 | 47.66 | 67.80 | 0.59 | 660.43 |
| 3 | 0.83 | 0.85 | 49.72 | 730.49 |
| 4 | 7.35 | 12.60 | 302.24 | 35.91 |
| 5 | 0.05 | 0.06 | 0.73 | 950.96 |
| 6 | 0.39 | 0.44 | 1.08 | 993.30 |
| 7 | 0.85 | 1.26 | 38.06 | 180.36 |
| 8 | 73.49 | 330.08 | 221.56 | 0.47 |
| 9 | 374.98 | 494.56 | 513.39 | 1864.26 |
| 10 | 20.38 | 27.16 | 112.71 | 888.73 |
| 11 | 9.44 | 10.55 | 21.01 | 962.26 |
| 12 | 3.08 | 3.72 | 29.59 | 763.64 |
| 13 | 119.06 | 133.61 | 316.59 | 1594.00 |
| 14 | 165.58 | 247.63 | 131.70 | 1.88 |
| 15 | 0.00 | 0.00 | 18.95 | 699.02 |

## Extra: Scripts and results from bootstrapping analyses

n_boot=10000; % 10000 bootstrap samples

W=[];

Unexplssq=[];

RMSE=[];

n=58;

rng(1); % set seed for reproducibility

for i=1:n_boot

bs=ceil(rand(n,1)*n); % bootstrap sample

Data_bs=Data(bs,:);

y_mean_bs=mean(Data_bs,1)'; %y_mean contains averages over experts

Xp=X(:,4:5);

b_bs=Xp\(y_mean_bs-1.98*X(:,6));

% collect bootstrap solutions

W(:,i)=b_bs;

% disp('proportion unexplained sum of squares')

UnexplssqRegr(i)=ssq(y_mean_bs-1.98*X(:,6)-Xp*b_bs)/ssq(y_mean_bs);

% disp('Root mean squared error of estimation')

RMSERegr(i)=sqrt(ssq(y_mean_bs-1.98*X(:,6)-Xp*b_bs)/15);

% disp('proportion unexplained sum of squares')

UnexplssqvdE(i)=ssq(y_mean_bs-PvdE*100)/ssq(y_mean_bs);

% disp('Root mean squared error of estimation')

RMSEvdE(i)=sqrt(ssq(y_mean_bs-PvdE*100)/15);

% disp('proportion unexplained sum of squares')

Unexplssqsd_norm(i)=ssq(y_mean_bs-sd_norm'*100)/ssq(y_mean_bs);

% disp('Root mean squared error of estimation')

RMSEsd_norm(i)=sqrt(ssq(y_mean_bs-sd_norm'*100)/15);

end;

>> disp('Regression');

[b_lo,b_up]=percentile95(W')

[U_lo,U_up]=percentile95(UnexplssqRegr')

[R_lo,R_up]=percentile95(RMSERegr')

disp('Van der Eijk');

[U_lo,U_up]=percentile95(UnexplssqvdE')

[R_lo,R_up]=percentile95(RMSEvdE')

disp('SD_norm');

[U_lo,U_up]=percentile95(Unexplssqsd_norm')

[R_lo,R_up]=percentile95(RMSEsd_norm')

**Regression**

b_lo =

0.9307 1.1929

b_up =

1.2000 1.5058

U_lo =

0.0218

U_up =

0.0555

R_lo =

6.1091

R_up =

9.4851

**Van der Eijk**

U_lo =

0.0541

U_up =

0.1164

R_lo =

9.5914

R_up =

13.6543

**SD_norm**

U_lo =

0.3558

U_up =

0.6159

R_lo =

25.2321

R_up =

30.6286

**Esteban and Ray measures (RMSE’s with CIs for ER with alpha 0, 0.8, 1, 1.6)**

Root mean squared error of estimation, alpha =0

ans =

96.2151

R_lo =

93.7073

R_up =

98.7676

Root mean squared error of estimation alpha =0.8

ans =

14.7493

R_lo =

12.4807

R_up =

17.2189

Root mean squared error of estimation alpha = 1.0

ans =

14.2972

R_lo =

12.9023

R_up =

16.0196

Root mean squared error of estimation alpha = 1.6

ans =

25.8552

R_lo =

24.2660

R_up =

27.6137

1. Although we cannot imagine how it would have any adverse effects, for completeness sake we want to mention that we did this kind of checking only *after* we found a strange result in the regression analyses. We found extreme and negative regression weights for the expert no. 37, and then verified whether something strange was going on here. Inspecting the scores, we realized that these were way too low, and this actually inspired us to inspect test scale usage of all experts.. [↑](#footnote-ref-1)
2. The reasoning is as follows. Let us assume that we have b_2_=1.98 and b_3_=1.98. We can start out from the situation where we have 50 scores of 1 and 50 scores of 5, which yields 4900 pairs of difference of 4 points and *f_2b_*=0, *f_3_*=0 and *f_4_*=50.51, yielding *P*=100. Now suppose we have 49 1s and 51 5s, then we have fewer pairs of differences of 4 points (49×51×2-100=4898), and hence *f_4_* will become smaller than 50.51, and *P* will become smaller than 100. Indeed, the number of differences is computed as *k*(100-*k*)-100, which has its maximum if *k*=50, as can be derived by elementary calculus. Now suppose we have 3 different scores, e.g., 30 1s, 20 2s and 50 5s, but still with 50 scores on both sides of neutral. Then the number of pairs with scores on both sides of neutral will jointly still be 4900, but now f_4_=30×50×2/9900×100=30.30 and f_3_=20×50×2/9900=20.20. Because the weights all equal 1.98, now *P* will be 1.98× (30.30+20.20)=100. In other words, for equal weights, we would have *P=*1.98f_2b_+1.98f_3_+1.98f_4_ = 1.98(f_2b_+f_3_+f_4_), so this expression simply takes 1.98 times the percentage of pairs of scores that are on both sides of neutral. So clearly the value of *P* then does not change if the values of fs are modified, but remain on the same side of neutral. Alternatively, if scores are modified such that they no longer are on the same side of neutral they must decrease the number of pairs with scores on both sides of neutral. Hence, when taking weights all equal to 1.98, the maximum is still 100. And as soon as one of the weights is taken lower than this, obviously the value of *P* can only decrease. This demonstrates that if the weights are required not to exceed 1.98, *P* will at most be 100. [↑](#footnote-ref-2)
3. We did the same analysis without distribution 3, and then found b= [1.0625 ; 1.3497], which suggests that the presence of distribution 3 does not seriously impact 3 in this analysis; because 3 does seem a valid representative of nonpolarized cases, we prefer to keep it in our analyses. [↑](#footnote-ref-3)
4. In all bootstrap analyses we used bootstrap samples of experts *with all their scores* because scores are dependent within experts. See appendix for scripts. [↑](#footnote-ref-4)
5. We should ensure that the highest possible value will not be above 100, and likewise, the lowest possible value cannot be negative. The latter will be attained if all weights are nonnegative, but how to attain the former is a more complex matter. To see this, suppose *b*_1_, *b*_2a_, *b*_2b_, and *b*_3_ would all equal 1.5 (hence clearly below 1.98), then a score distribution of 20, 20, 20, 20, 20 for scores 1,..,5, respectively, would lead to a *P* =125.1 (7200/9900×100×1.5+800/9900×100×1.98), clearly above 100. So, even a distribution which clearly is not polarized would with such weights lead to a *P* value higher than 100. This is caused in particular by the high value of *f*_1_ for such score patterns, and hence, the weight for *f*_1_ in a polarization measure should be quite low indeed. In practice, one can expect this to actually happen. [↑](#footnote-ref-5)
6. Alternatively, we could state that P should be 0 when the scores are all 3, 4 or 5, or all 1,2, or 3, hence all on one side of the scale. It then follows that for P=0 it must hold that f2b=f3=f4=0, because then there are no pairs of scores like (2,4), (1,4) or (1,5). Hence in that case P=b0*f0+ b1*f1+b2a*f2a, which must equal 0 as stated above. For a particular case, this can be attained for various sets of weights b0, b1 and b2a. However, as this must hold for *all* cases where f2b=f3=f4=0, it is easy to see that for this it is required that b0=b1=b2a=0 (based on having more equations than unknown). This brings us back to the first case we studied, where only f2b, f3 and f4 are used as predictors. [↑](#footnote-ref-6)
